# Chiral Symmetry Breaking in Colloidal Metal Nanoparticle Solutions by Circularly Polarized Light

**DOI:** 10.1021/acsnano.4c09349

**Published:** 2024-10-05

**Authors:** Monika Ghalawat, Daniel Feferman, Lucas V. Besteiro, Wanting He, Artur Movsesyan, Alina Muravitskaya, Jesus Valdez, Audrey Moores, Zhiming Wang, Dongling Ma, Alexander O. Govorov, Gil Markovich

**Affiliations:** † School of Chemistry, Tel Aviv University, Tel Aviv 6997801, Israel; ‡ CINBIO, University of Vigo, Vigo 36310, Spain; § Énergie Matériauxet Télécommunications, Institut National de la Recherche Scientifique (INRS), 1650 Bd Lionel-Boulet, Varennes, Quebec J3X 1P7, Canada; ∥ Department of Physics and Astronomy and Nanoscale and Quantum Phenomena Institute, Ohio University, Athens, Ohio 45701, United States; ⊥ Institute of Fundamental and Frontier Sciences, University of Electronic Science and Technology of China, Chengdu 610054, China; # Facility for Electron Microscopy Research (FEMR), McGill University, 3640 University Street, Montréal, Quebec H3A 037, Canada; ∇ Centre in Green Chemistry and Catalysis, Department of Chemistry, McGill University, 801 Sherbrooke Street West, Montréal, Quebec H3A 0B8, Canada

**Keywords:** nanoscale chirality, plasmonic nanoparticles, circular dichroism, circularly polarized light, galvanic replacement reaction

## Abstract

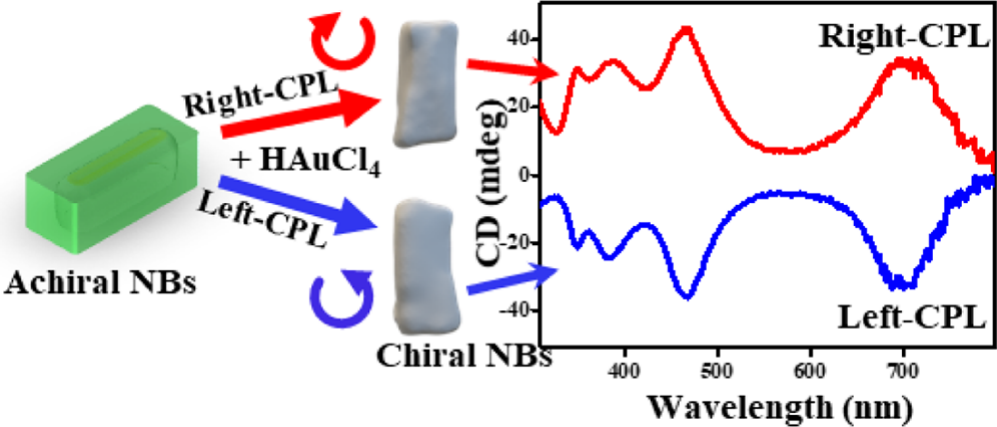

Shape symmetry breaking in the formation of inorganic nanostructures is of significant current interest. It was typically achieved through the growth of colloidal nanoparticles with adsorbed chiral molecules. Photochemical processes induced through asymmetric plasmon excitation by circularly polarized light in surface immobilized nanostructures also led to symmetry breaking. Here, we show that chiral symmetry breaking can be achieved by randomly rotating gold@silver core–shell nanobars in colloidal solution using circularly polarized illumination, where orientational averaging does not eliminate the symmetry breaking of an asymmetric plasmon-induced galvanic replacement reaction. Different morphological effects that are produced by circularly vs linearly polarized light illumination demonstrate the intricate effect of light polarization on the localized plasmonic-induced photochemical response. The essential features of this symmetry breaking, such as illumination wavelength dependence, were reproduced by simulations of circularly polarized light-excited-plasmon-induced hot-electron generation as the source for asymmetric metal deposition. The symmetry breaking becomes smaller in more symmetric geometrical shapes, such as triangular nanoprisms and nanocubes, and down to zero in spherical ones. The degree of symmetry breaking rises when the nanobars are immobilized on a substrate and illuminated from a single direction.

## 1 Introduction

Breaking the symmetry in the shape of colloidal metal nanostructures has been a long-standing challenge. Relatively simple processes of symmetry breaking in face-centered cubic metals, such as gold nanoparticles (NPs), use certain surfactant molecules to cause the directional growth of spherical seed NPs into nanorods (NRs).^1,2^ In recent years, more elaborate symmetry breaking was reported, leading to the formation of nanostructures with chiral shapes.^3,4^ These methods involve the interaction of chiral molecules with the surface of metal NPs to cause asymmetric (chiral) metal deposition. The interest in obtaining strongly chiral noble metal nanostructures was mainly focused on increasing the magnitude of chiroptical activity by orders of magnitude compared to that of chiral molecules. This is possible due to the increase of chirality scale from the Angstrom scale of asymmetric carbon bonds to a chiral twist pitch of the order of ∼100 nm,^5^ approaching the wavelength of visible light, and due to the delocalized free electrons in the metal nanostructure, which provide the strong optical response of surface plasmon resonance.^6−10^

Plasmon excitation has a strong influence on the shape evolution of silver NPs.^11,12^ A particularly striking finding was that of Sastry and coworkers, where illumination at different wavelengths of triangular silver nanoprisms (TNPs), while reacting with gold ions to obtain partial galvanic replacement reaction (GRR) of silver by gold, led to different morphologies of the gold-decorated silver TNPs.^13^

Circularly polarized light (CPL) is a form of chiral “disturbance,” which could affect chemical reactions and break symmetry in chiral molecular synthesis.^14,15^ However, such symmetry breaking would normally be very small, similar to the scale of the molecules’ optical activity, leading to tiny enantiomeric excess of the product molecules.^16^ In contrast to molecules, exciting asymmetric plasmon resonance modes in metal nanostructures could cause symmetry breaking in plasmonic particles of the scale of ∼100 nm, which could exhibit much larger optical activity relative to small molecules.^5^ In the work of Saito and Tatsuma, gold nanocuboids anchored onto a TiO_2_-coated substrate were illuminated with CPL, which caused the localized oxidation of Pb^2+^ ions and consequent asymmetric deposition of PbO_2_ at opposite corners of the nanocuboids, giving rise to circular dichroism (CD) in the nanocuboids.^17^ Qiao et al. obtained similar effects for gold bipyramids,^18^ Ahn et al. demonstrated CPL-induced asymmetric polymerization patterns,^19^ and Xu et al. observed a combined CPL and chiral ligand effect on symmetry breaking.^20^ CPL was also used to affect NP assembly and obtain weak optical activity.^21,22^ A different type of chiral metal nanostructures was obtained using relatively high intensity light beams carrying orbital angular momentum.^23^ Govorov and coworkers have simulated this type of symmetry breaking based on hot-electron (HE) injection to metal ions at plasmonic hot spots.^24^ They demonstrated strong symmetry breaking and CD response of metal nanostructures on substrate surfaces induced by illumination from a single direction. They have also shown that weaker asymmetry and optical activity were obtained from CPL illumination of the nanostructures in colloidal solution, with orientation averaging of the illumination direction (averaging over three illumination directions).^25^ The magnitude of optical activity is usually expressed by the anisotropy factor, *g* factor = 2 × CD/Absorbance.

**Figure 1:**
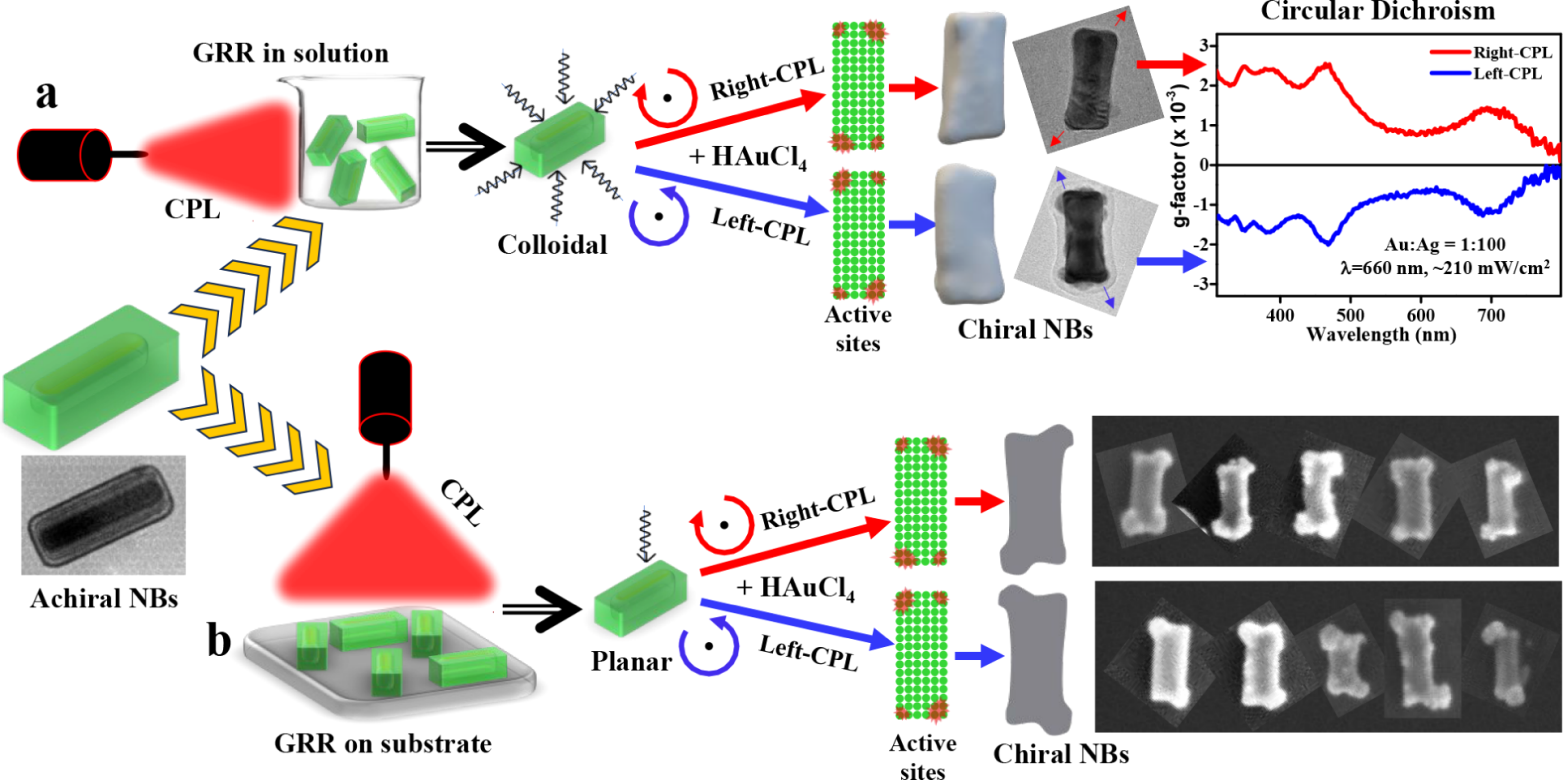
Scheme of two different routes to the transformation of achiral NBs into chiral ones. (a) Through GRR under CPL illumination of the colloidal NBs in solution with rotational averaging, displayed with the CD dissymmetry factor spectra obtained by right- and left-CPL illumination. (b) The same illumination experiment performed on surface immobilized NBs, leading to stronger shape asymmetry, as seen in several SEM images of NBs deposited on a silicon substrate and undergone GRR with right- and left-CPL illumination.

In the present work, we experimentally demonstrate chiral symmetry breaking of Au@Ag core–shell nanobars (NBs) dispersed in solution through CPL illumination with orientation averaging, while the particles undergo partial GRR, as schematically illustrated in Figure 1. Detailed studies are conducted to understand the significance of silver shell thickness, Au:Ag atomic ratio used for the GRR, illumination wavelength, and the original NP shape. It is found that, as expected, the asymmetry of the shapes becomes stronger when the NBs are immobilized on a substrate and illuminated from a single direction, perpendicular to their long axis.

## 2 Results and Discussion

### 2.1 NB Synthesis and Properties

The Au@Ag NBs were prepared by the surfactant-controlled overgrowth of silver on gold NRs. UV/vis/NIR spectroscopy and scanning electron microscopy (SEM) were used to characterize the Au NRs as shown in Figure S1. The NRs exhibit an extinction peak of the transverse plasmon resonance mode at 510 nm and a near-infrared longitudinal mode at 842 nm. The NRs were about 77 ± 3 nm in length and 17 ± 2 nm in width. They were used as the cores for coating with a silver shell of varying thickness. The added silver ion concentration was varied to obtain several types of NBs with different silver shell thickness, between ∼11–37 nm, labeled Samples 1–6 (see Table S1). The growth of the silver shell on the Au NRs occurred primarily at the lateral facets, as can be seen on the left side of Figure 1 (see also Table S1), reducing the aspect ratio of the NBs with silver shell thickening.

**Figure 2:**
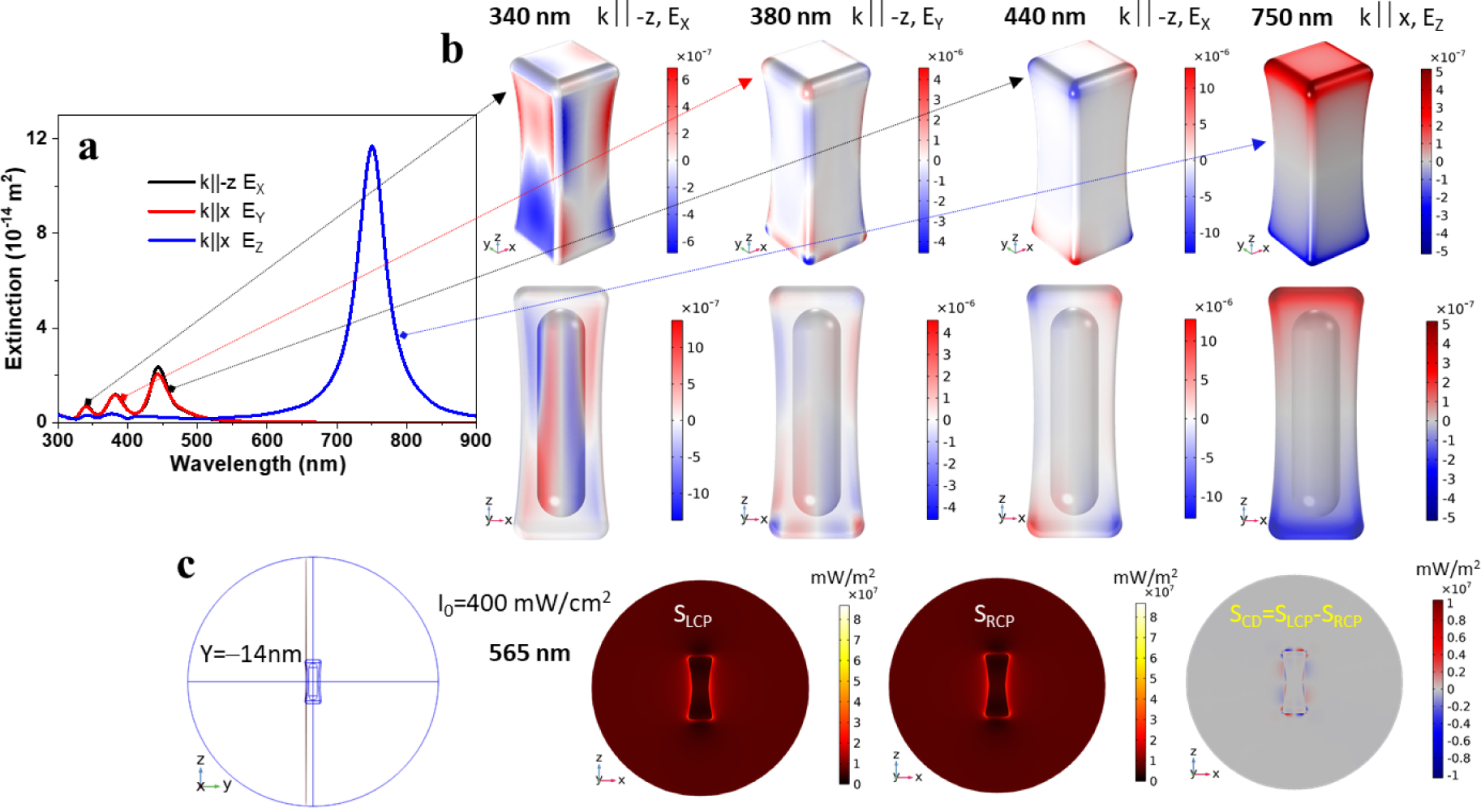
(a) Simulated extinction spectra of the Au@Ag NBs for several illumination conditions: light incidence (*k* vector) along *x* or *z* directions (*z* is the long axis) and linear polarization along *x*,*y*,*z* axes. Four different plasmon resonance peaks can be observed. The sizes used in the simulation: Au NR core: 74 × 17 nm, Ag shell: 86 × 40 nm (35 nm width at the center). (b) Simulated snapshots of surface charge density distributions (real component) following excitation of each of the resonances and varying incidence direction and linear polarization axis (indicated at the top). The bottom row shows the surface charge distribution also at the Au/Ag interface by making the silver shell semitransparent. The resonance at 340 nm is called silver “slab” resonance since it is primarily a dipole formed across the silver shell. The scales are in C/m^2^ units. (c) Simulated time- and illumination-direction-averaged absolute value map of the Poynting vector near the surface of the NBs following left-CPL (LCP) and right-CPL (RCP) illumination. The calculation was done for illumination wavelength of 565 nm, in between the two major, longitudinal, and transverse resonances. The left diagram shows that the presented data correspond to a cross-section (across *x*–*z* plane) at −14 nm along the *y*-axis with respect to the NB center. The right-most plot is a cross-sectional map of the difference in magnitude of the Poynting vector between the LCP and RCP illuminated NBs, illustrating the asymmetry in the local hot spot distribution between the two circular polarization excitations. The particle shape was made slightly concave to approximate the initial stage of the GRR, where Ag is slightly dissolved from the center of the NBs.

Four plasmon resonance peaks, unique to cuboid silver and gold-core/silver-shell NBs,^26−28^ can be seen in the extinction spectra of the NBs for Samples 1 to 4, before merging of the longitudinal and transverse resonance modes into a single peak for the nearly cubic samples 5 and 6 (Figure S2). Figure 2a (and Figures S3–S5) displays the simulated plasmon resonance features for these four plasmon resonances. The longitudinal dipolar plasmonic mode (experimental 685 to 731 nm, simulated 750 nm) is the energetically lowest resonance mode. The transverse dipolar mode excited perpendicular to the long axis of the NBs appears below 500 nm. The appearance of a sharp transverse mode (between 443 to 484 nm) indicates the formation of NBs. The plasmon resonance mode at ∼380 nm is characteristic of edge plasmons at silver nanocubes and seems to have also a higher-order multipolar character.^29^ The shortest resonance mode is a dipolar silver “slab” resonance, oscillating primarily across the silver shell, as can be seen in the lower panel of Figure 2b.

We have also studied the nature of plasmon resonances under CPL illumination, as shown in Figure 2c (and Figures S6–S10), using the electromagnetic simulations. The simulations were performed on slightly concave NBs in order to imitate the situation at the beginning of the GRR process where silver is dissolved from the center of the long faces of the NBs. It can be seen that with directional illumination (Figures S6–S9) the asymmetry level in localized surface hot spots is larger than the orientationally averaged case (Figure S10). In addition, it seems that the excitation near the transverse resonance peak wavelength (457 nm) is more effective in symmetry breaking (in terms of peak pointing vector asymmetry between right-CPL and left-CPL excitation) compared to excitation to the blue side of the longitudinal resonance wavelength (682 nm), or between the resonances, at 565 nm (Figure 2c). Nevertheless, for both excitations between the longitudinal and transverse resonances (565, 682 nm), while having lower absolute values of peak asymmetry in the averaged pointing vector distribution (S_CD_ = S_LCP_ – S_RCP_), they have comparable relative values with respect to either the S_LCP_ or S_RCP_ peak magnitudes.

### 2.2 GRR Under Illumination

The silver shell of the NBs reacted with aqueous AuCl_4_^–^ ions to achieve a spontaneous GRR between these two species. This reaction was monitored by observing the changes in the extinction spectrum of the NBs, as the peak wavelengths of the plasmon modes are particularly sensitive to changes in NB composition, shape, size, and local dielectric environment.

**Figure 3:**
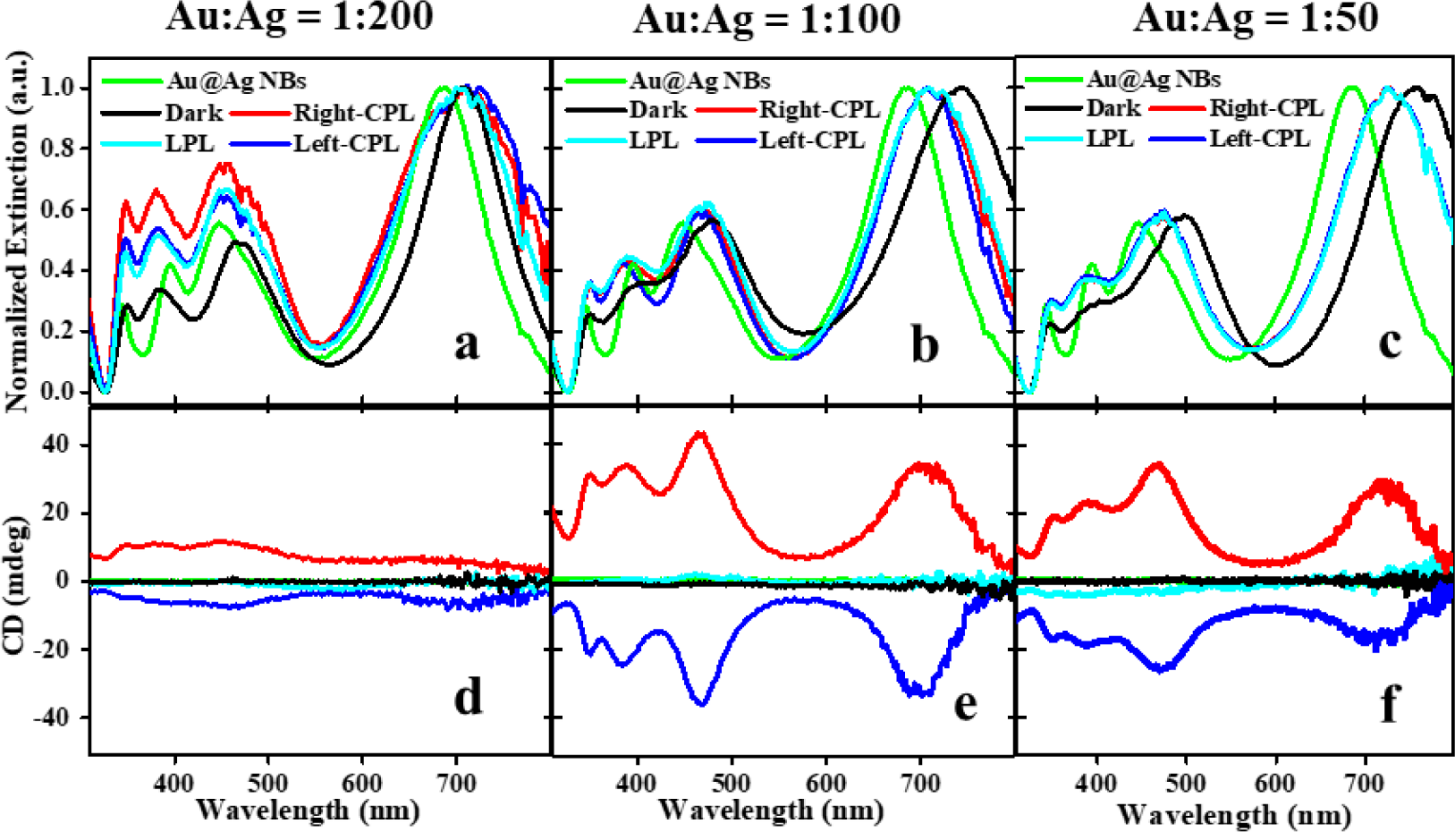
Extinction and CD spectra of NBs post GRR under CPL illumination. (a–c) Normalized extinction spectra of the original Au@Ag NBs (Sample 2): NBs after GRR in the dark, after GRR under LPL illumination, and after GRR under left- and right-CPL illumination at 660 nm with the indicated Au:Ag atomic ratios. (d–f) The corresponding CD spectra of the same samples.

Figure 3 shows the results of the GRR experiments conducted with Sample 2 under 660 nm (∼210 mW/cm^2^) CPL illumination with Au:Ag molar ratios in the range of 1:50 to 1:200. This molar (atomic) ratio represents the ratio of gold ions added for the GRR reaction relative to the number of silver atoms used for the coating of the Au NRs to form the NBs. Continuous illumination (for 5 min) using either left-CPL or right-CPL polarizations was compared to reference cases of dark and linearly polarized light (LPL) illumination. All were done under stirring, where about half of the solution is illuminated with the collimated beam (of ∼1 cm^2^ cross section) and solution concentration was tuned to achieve absorbance value of ∼1.1 (at the longitudinal plasmon resonance peak) at the center of the round vial used for the reaction.

Fairly strong CD lines of opposite polarity appeared for the GRR under right- and left-CPL illumination experiments, where the strongest CD effect was observed for the 1:100 Au:Ag atomic ratio (see also Figures S11 and S12) resulting in peak dissymmetry factor,  (Figure 1a). This is a modest value for the optical activity of strongly chiral plasmonic nanostructures, but significantly larger than most reported values involving achiral colloidal metal nanostructures interacting with chiral molecules,^30^ or assembled into chiral structures by CPL.^21,22^ It should be noted that the reduction of each Au(III) ion requires 3 Ag atoms to be oxidized; hence, a 1:100 Au:Ag atomic ratio would achieve 1:33 Au–Ag atomic replacement for a process with 100% redox reaction yield. Zero CD was consistently observed for dark and LPL illumination for all samples. Due to the achiral geometry of the original NBs, they likewise displayed zero CD. Throughout this work, in experiments that yielded significant CD signals (few millidegrees and up), illumination with right-CPL always produced positive CD and with left-CPL always produced negative CD lines.

When the Au:Ag atomic ratio was further increased to 1:20, the NBs’ CD peak decreased to nearly 6 mdeg, as seen in Figure S11. Relatively high Au:Ag GRR atomic ratio (for example 1:10) produced nearly zero CD signals when exposed to CPL illumination at 660 nm during GRR, probably due to greater radical NB shape changes. Conversely, when the ratio was decreased to 1:200, for example, the degree of morphological change became too small to obtain significant shape dissymmetry. The optimal CD response (as a consequence of geometrical chirality) is a complex function of many parameters. In the present case, it was around a Au/Ag ratio of 1:100 (for maximal power of 660, 565 nm illumination) probably due to matching of GRR and light-induced plasmonic photochemical rates. We have seen that with lower illumination intensity, the optimal ratio goes to lower Au:Ag ratios (Figure S13).

The SEM images of the products of GRR in the dark vs under LPL photoexcitation conditions from NBs with different silver shell thicknesses reveal stark differences (Figures S14–S16). In the dark, GRR leads to formation of deposited Au nanocages (Au ions reduced by the silver shell), or nanorattles, with the original gold NRs trapped inside the nanocages, within the voids left by the dissolved Ag shells. Here, the GRR began with a pinhole at the surface of the NBs, and as the reaction proceeded (with increasing gold ion concentration), the pinhole evolved into a void on the flat face of the NBs, finally resulting in an Au nanocage containing a NR (Figure S17a). In contrast, GRR under photoexcitation of the NBs resulted in concave surfaces, evolving to an overall bone-like shape as gold ion concentration is increased (Figure S17b).

To determine the significance of silver shell thickness, the same illuminated GRR reactions were carried out with NBs with slightly thinner (Sample 1) and slightly thicker (Sample 3) silver shells, relative to Sample 2, while maintaining a constant Au:Ag GRR ratio (1:20). The CD signal for Sample 1 decreased relative to Sample 2, and almost disappeared for Sample 3 (Figure S18). It can therefore be concluded that there is an optimal thickness of the silver shells for inducing an asymmetry in the NBs by CPL illumination. Since the NBs of Sample 2 exhibited the strongest CPL-induced symmetry-breaking effects, the rest of the paper will deal with this type of NBs only. Transmission electron microscopy (TEM) image analysis shows that as-prepared NBs (Sample 2) have an average width of 38 ± 4 nm and length of 88 ± 5 nm (Figure S16a). The coating thickness on the sides of the NBs is, therefore, 10 ± 4 nm.

### 2.3 Study of the Shapes of NBs After GRR Under Illumination

**Figure 4:**
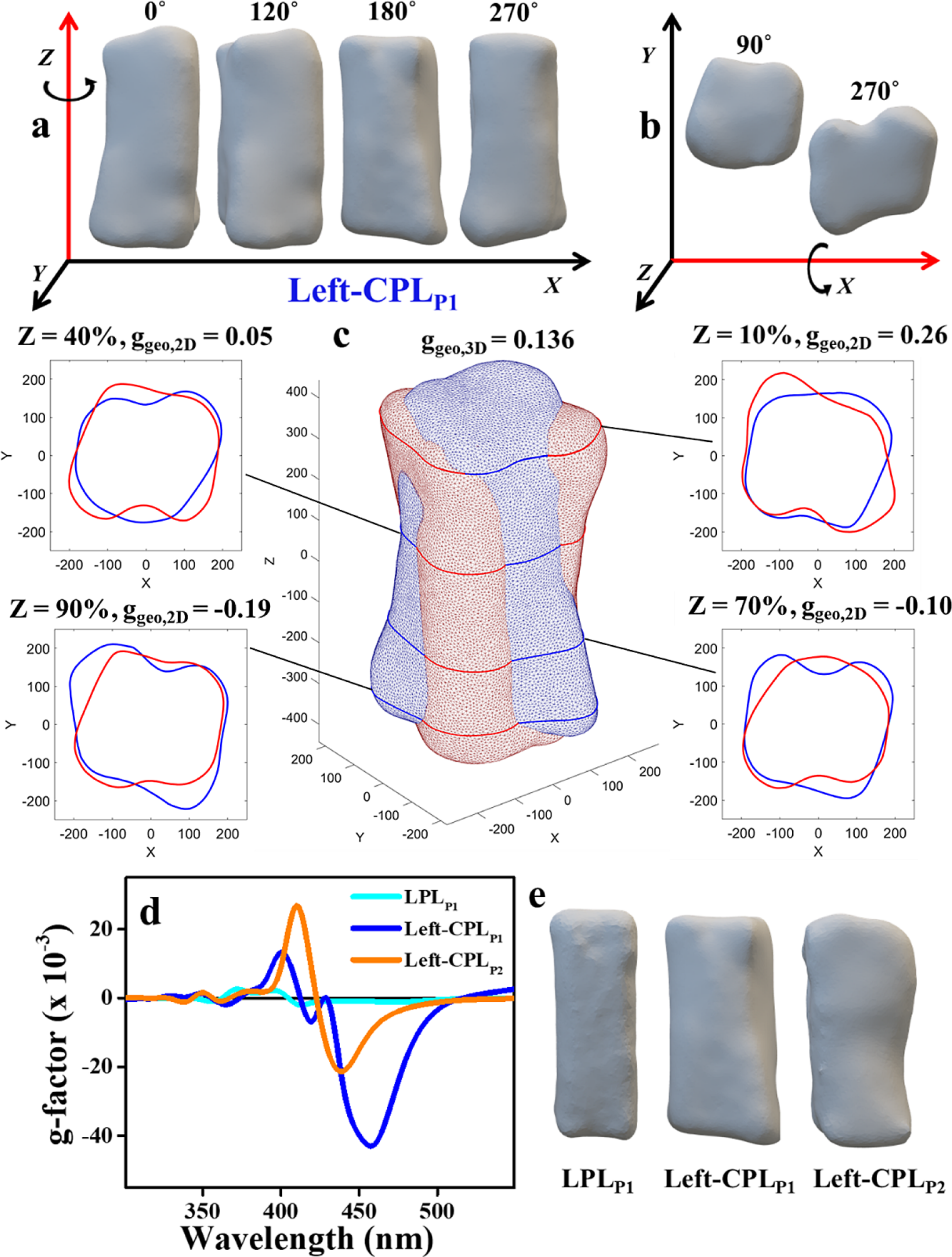
Electron tomography results on post-CPL-illuminated-GRR NB morphology. (a) Snapshots of rotated (around the long axis) projections of an electron tomography 3D reconstruction of one of the left-CPL illuminated GRR experiments using 660 nm illumination with Au:Ag atomic ratio of 1:100 (labeled: Left-CPL_P1_). (b) Projections of the two ends of the same NB. (c) Geometrical analysis of the chirality of electron tomography 3D reconstructed shape of the same NB. The NB shape, shown in blue, was first displaced to coincide its center of mass with the origin, and the two largest eigenvectors of the inertia matrix of the NB were aligned with the Z- and *Y*-axes. Then the body was mirrored across the *Z*–*Y* plane with the resulting geometry shown in red. Four cross sections of the shape parallel to the *X*–*Y* plane are shown; demonstrating the asymmetry at four different Z-positions, with the 2D normalized chirality parameter value in each of them. The 3D object’s normalized chirality parameter’s value (*g*_geo,3D_) is also presented (see definition in the Methods section). (d) The simulated CD g-factor spectra of the 3D reconstructed shapes of three different individual particles after GRR under LPL (LPL_P1_) and left-CPL (Left-CPL_P1_ and Left-CPL_P2_) illumination conditions. (e) Electron tomography 3D reconstruction snapshots of the same NBs used for the simulations.

Electron tomography characterization performed on several single NBs after GRR under illumination with CPL provides information on the nature of their 3D chiral structure (see Figures 4a,b,S19a–d and S20). A chiral bone-like shape is observed for all particles, with some randomness in the shape details.

In order to assess geometric chirality, we analyzed the overlap between the volumes of an NB and its mirror image after aligning the NB along two of its principle moments of inertia and reflecting across a plane defined by the two axes. The result of this analysis of one of these NBs, labeled Left-CPL_P1_, is shown in Figure 4c and another particle is analyzed in Figure S19e. The asymmetric features are mostly arranged as bulbs along the edges of the NB. The volume integration over the two enantiomers, only partially overlapped, yielded a normalized geometrical dissymmetry value, *g*_geo_,_3*D*_ = 0.136 (see definition in the Methods section) for the nonoverlapping volume fraction; i.e., the chiral fraction of the shape is about 7% of the total volume. However, this value may vary significantly between individual NBs, as in the example of the second analyzed NB (Left-CPL_P2_, Figure S19e), *g*_geo_,_3*D*_ = −0.015. As the measured CD spectra are an average of all NBs and they showcase a well-defined CD polarity and shape handedness, especially for samples Left-CPL_P1,P2_, we can reliably conclude that there are enough asymmetrical NBs to cause a macroscopic effect. Figure 4d shows the simulated CD dissymmetry factor for three different reconstructed 3D structures from three individual NBs, shown in Figure 4e. While the two NBs analyzed from the left-CPL illuminated sample exhibited significant and qualitatively similar simulated CD spectra with peak g-factors of nearly −0.04 and −0.02, the simulated spectrum for the reconstructed structure of the NB after GRR under LPL illumination (control sample, LPL_P1_) was nearly zero, as expected. There was not enough numerical precision for reliably calculating the g-factors at wavelengths greater than 550 nm.

We believe that the averaging over the distribution of different individual particles’ CD spectra may be the cause for the experimental CD signal being of single polarity. A hint for this is given by the combination of the simulated spectra of the two Left-CPL particles, where a negative line at 440–450 nm should be the dominant CD feature after the two spectra.

**Figure 5:**
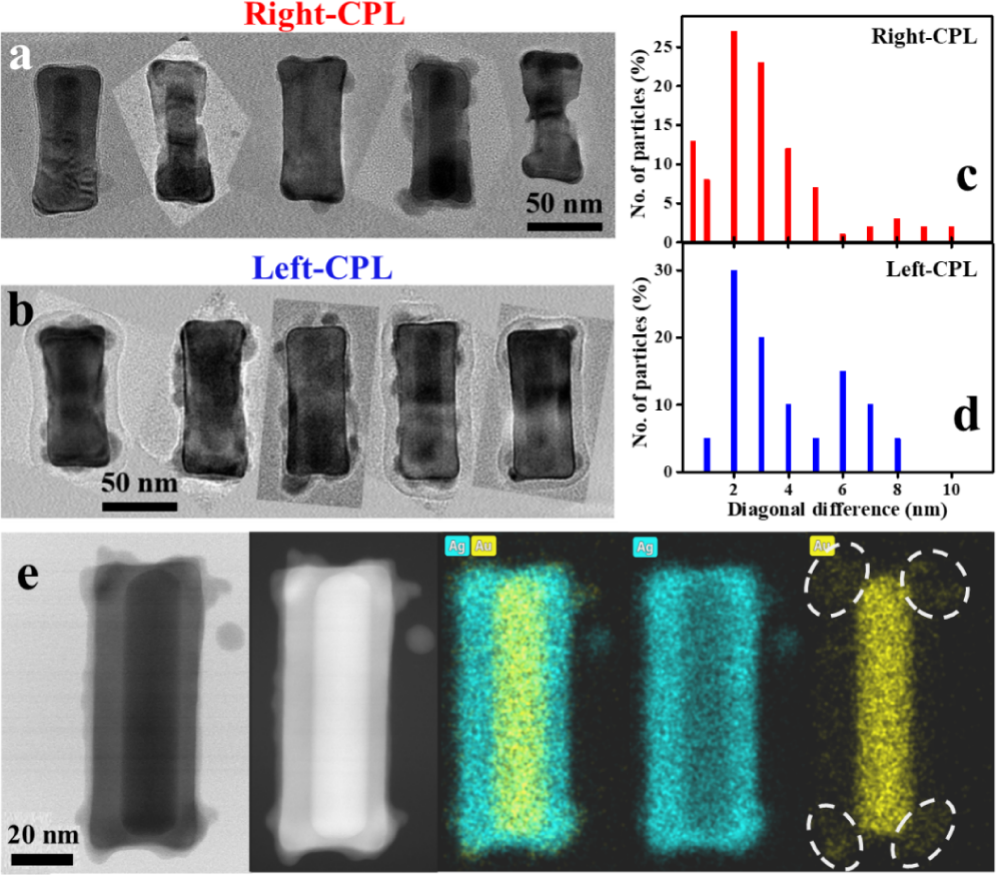
TEM images of the NBs post GRR under CPL illumination. (a,b) Bright-field TEM images of several NBs from the right and left-CPL illuminated GRR experiments using 660 nm illumination with Au:Ag atomic ratio of 1:100. (c,d) Histograms of the distribution of differences between the two diagonals in ∼50 NBs as an indicator of 2D asymmetry of the shape for the corresponding samples. (e) STEM bright field, dark-field, and EDS elemental mapping of silver and gold in one NB post-GRR with right-CPL illumination. The dashed ellipsoids mark a higher concentration (relative to the facets) of gold deposited during GRR at the corners of the NB.

While 3D shape tomographic reconstruction and shape analysis of single particles are highly time-consuming and difficult to perform on many NBs, analysis of the 2D projection in regular TEM imaging can provide statistically meaningful information about the shape asymmetry. Hence, we measured the difference between the two main diagonals and the two end widths in the TEM images of 50 NBs after GRR under CPL vs LPL illumination at 1:100 Au:Ag atomic ratio (Figures 5a–d and S21). While the most frequent diagonal difference for LPL illumination is ∼1 nm (which is about 1% of the diagonal length) and the maximal difference is 2 nm, for CPL illumination, the most frequent diagonal difference is about 2–3 nm and maximal difference is 8–10 nm.

Moreover, in the TEM images of the original Au@Ag NBs, for each NB, the difference between the widths of its two ends is nearly zero, up to ∼1 nm. After GRR under LPL illumination, this value became ∼2 nm on average and reached a maximum of 5 nm. The average end-width difference in the presence of CPL illumination at 660 nm with an Au:Ag atomic ratio of 1:100 is 3 nm and the maximum value reaches 12–15 nm (Figure S21). Hence, CPL illumination significantly increased the level of shape asymmetry in the GRR of the NBs relative to LPL and dark. This further strengthens the preliminary impression of the shape difference between the LPL and two Left-CPL particles shown in Figure 4e.

The average maximal width value in the original Au@Ag NBs is 38 ± 4 nm. It increases to 40 ± 5 nm under GRR with LPL/CPL illumination (Figure S22). This was further investigated by performing STEM-EDS elemental mapping after GRR under right-CPL illumination with a 1:100 Au:Ag atomic ratio, as shown in Figures 5e and S23. There is a greater concentration of gold around the corners of the NBs compared to their central parts, as highlighted by the ellipsoids in the Au map (see quantification in Figure S23). Similar effects were observed in STEM-EDS maps of NBs exposed to LPL (Figure S24). The silver map also shows that there is an asymmetric widening of the silver distribution at the edges. Occasionally, especially for relatively higher Au:Ag atomic ratios, the corners of the NBs appear to be the primary site of the redeposition of silver particles. Hence, it seems that some of the dissolved silver ions were asymmetrically redeposited at the NB corners, together with deposited gold ions under the influence of the plasmonic excitation at 660 nm. It should be stressed that quantification of the Au:Ag atomic ratio at the corner volumes (Figure S23) shows that there is only 3–8% atomic fraction of gold. This ratio diminishes to about 1–1.5% away from the corners. Hence, it seems that the main plasmonic CD effect should come from the redistribution of silver in the shell.

### 2.4 Illumination Wavelength Dependence

**Figure 6:**
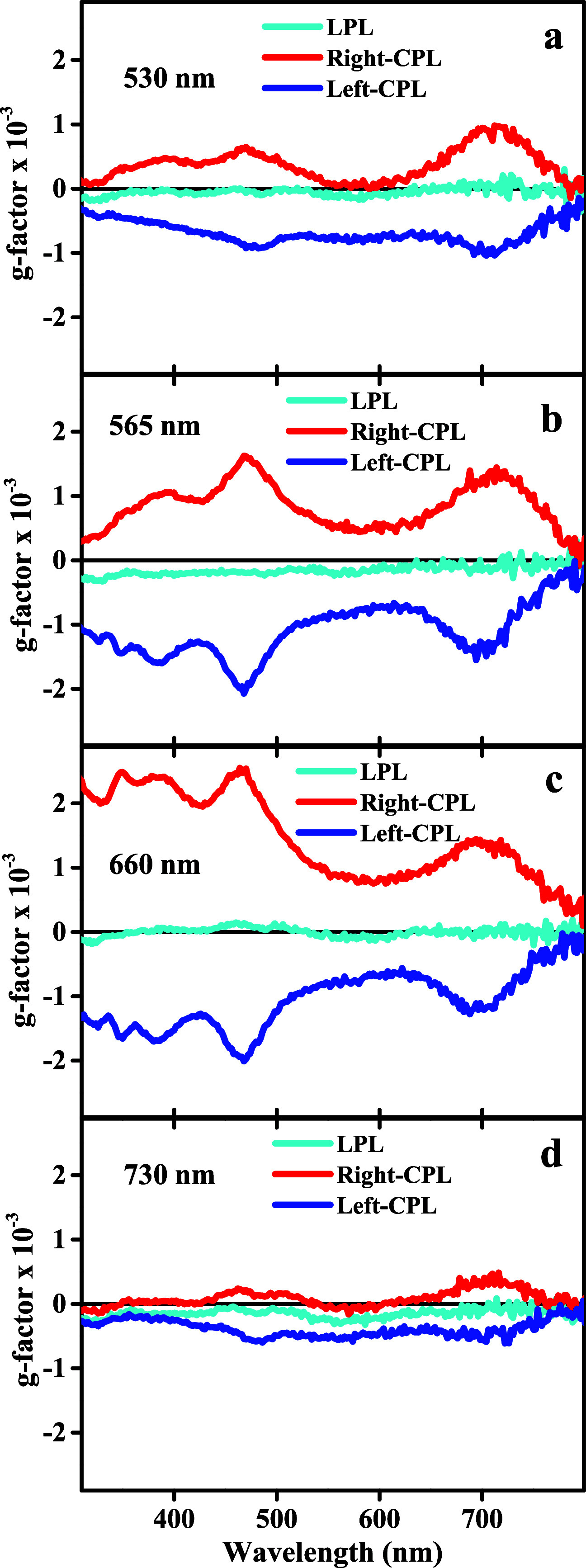
*g*-factor spectra of CPL- vs LPL-illuminated GRR in Au@Ag NBs with Au:Ag atomic ratio of 1:100, at different illumination wavelengths: (a) 530 nm, 100 mW/cm^2^; (b) 565 nm, 120 mW/cm^2^; (c) 660 nm, 210 mW/cm^2^; (d) 730 nm, 140 mW/cm^2^.

Figure 6 (and Figure S25) shows the results of GRR experiments performed with the Au:Ag atomic ratio of 1:100, under illumination at four different wavelengths from 530 to 730 nm. A wavelength dependence of the GRR within the red-green parts of the spectrum is observed. Here too, for all four wavelengths, nonzero, opposite polarity CD lines for the experiments with right and left-CPL illumination were observed, whereas for dark and LPL illumination, null CD was consistently observed. The statistics of the 2D geometrical asymmetry from the TEM images, measured for the different wavelengths of illumination (Figure S26), are in line with the 660 nm LPL and CPL-illuminated GRR. Figures S27–S30 show TEM and SEM images with many CPL-illuminated NBs, demonstrating the overall similarity of the resulting NBs, but with a variety of fine details.

Interestingly, the most effective wavelength for symmetry breaking seems to be 565 nm (*g*-factor comparable to 660 nm but achieved with almost half illumination intensity), which is located midway between the two major resonances. Hence, 565 nm illumination is probably the optimal wavelength for exciting asymmetric plasmon resonances due to out of phase excitation of a combination of the NBs’ transverse and longitudinal resonances with CPL.^31^

The illumination wavelength seems to affect the ratio of the longitudinal to transverse peak CD intensities (Figure S31a,c). It also seems that wavelengths shorter than the longitudinal plasmon resonance peak located at ∼690 nm are more effective in symmetry breaking than the 730 nm wavelength, which is located at the long-wavelength side of the peak. The CD spectrum of the 530 nm illuminated sample is lower than those of the samples illuminated by 565 and 660 nm. This could be due to its lower intensity (100 mW/cm^2^) as well as the spectral location far from the longitudinal plasmon resonance of the NBs. Additionally, a roughly linear relation between the intensity of the 660 nm illumination and the magnitude of the obtained CD signal of right-CPL illuminated GRR NBs was observed (Figure S31b,d). The relative intensities of the CD peaks at the two main resonances change with the illumination intensity. Hence, it seems that part of the wavelength dependence of the CD spectra is related to the strength of the plasmon excitation. Additionally, when the Au:Ag atomic ratio was reduced from 1:100 to 1:200 under right-CPL illumination at 530 nm, (Figure S32a,d) the intensity ratio of the two main CD peaks changed as well. In contrast to illumination at the more effective wavelengths of 565 and 660 nm, illumination at 730 nm produced stronger CD lines with GRR at 1:200 Au:Ag ratio, compared to 1:100 ratio (Figure S32c,f). Hence, it seems that for weaker excitation rates (either lower illumination intensity or wavelength out of the 600 nm regime) the symmetry breaking works better with slowing down of the GRR process through lowering the gold concentration.

**Figure 7:**
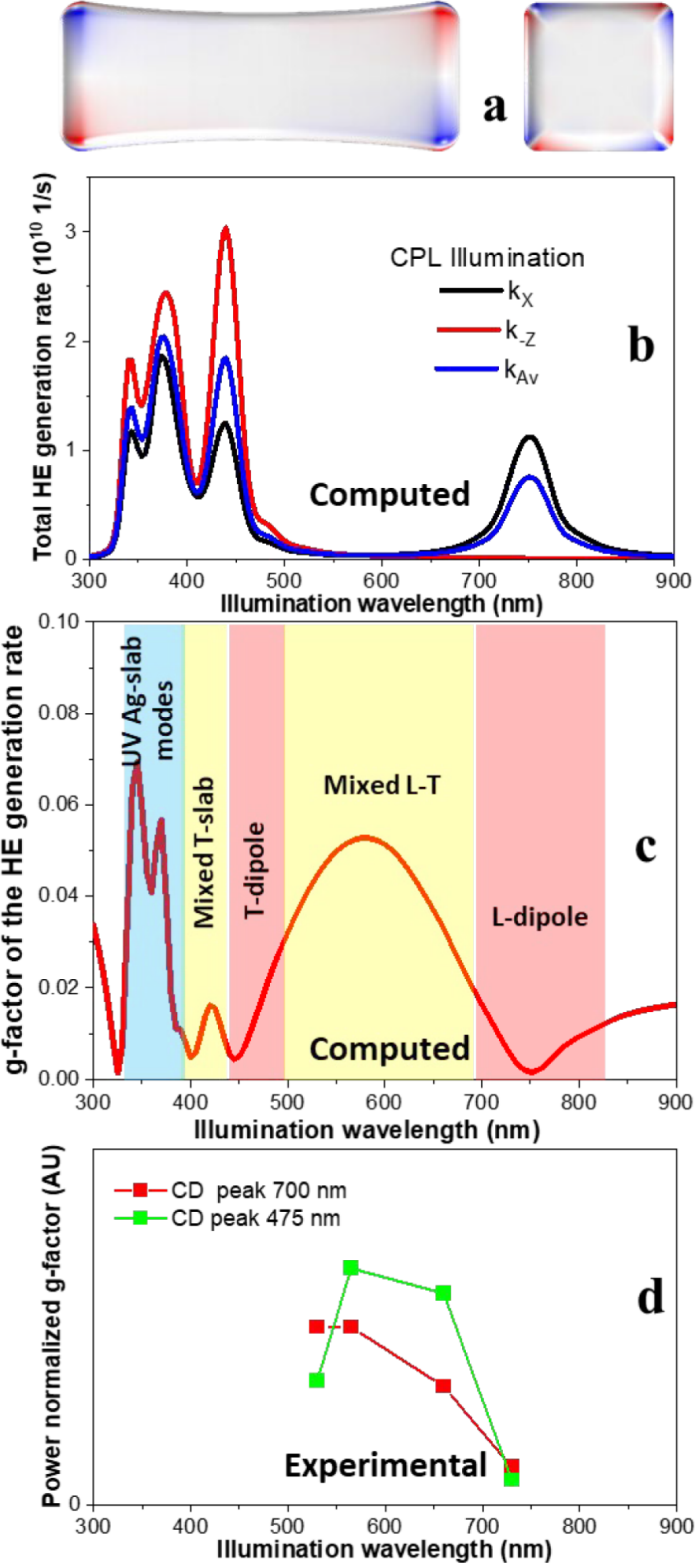
(a) Simulated NB surface map of the difference in HE generation rates between excitations by the two circular polarizations with averaged illumination direction. Illumination wavelength is 600 nm. (b) The spectral dependence of HE generation rates of the NBs computed by COMSOL for CPL illuminated NBs at orientation parallel to the long axis (*z*), perpendicular to it (*x*), and averaged over all directions. (c) Computed *g*-factor values for the HE generation rate integrated over the whole surface of the NBs, showing that the optimal wavelength for symmetry breaking is ∼600 nm. The different plasmon resonance ranges are highlighted with different background colors. (d) Experimental CD *g*-factors for the longitudinal (L-dipole) and transverse (T-dipole) resonance peaks as a function of the millumination wavelength. The peak *g*-factor values were normalized to the illumination intensities at the four wavelengths used for a proper comparison to the computed HE rates.

Figure 7b,c shows the calculated total rates of HE generation and HE generation rate *g*-factors for the surface HE maps (Figure 7a). The nonzero *g*-factor comes from the chiral patterns appearing at the surface of a NB when we compute the map of the HE in the solution setting (illumination orientational averaging). Interestingly, the maximum of the calculated HE *g*-factor appears in-between the strongest longitudinal (L-mode) and transverse (T-mode) resonances, indicating that the chirality requires a mixing between two modes. Whereas, a single pure dipolar mode carries smaller chirality. The power-normalized experimental g-factors from the Au@Ag NRs follow the same trend Figure 7d.

### 2.5 Effect of NP Shape

**Figure 8:**
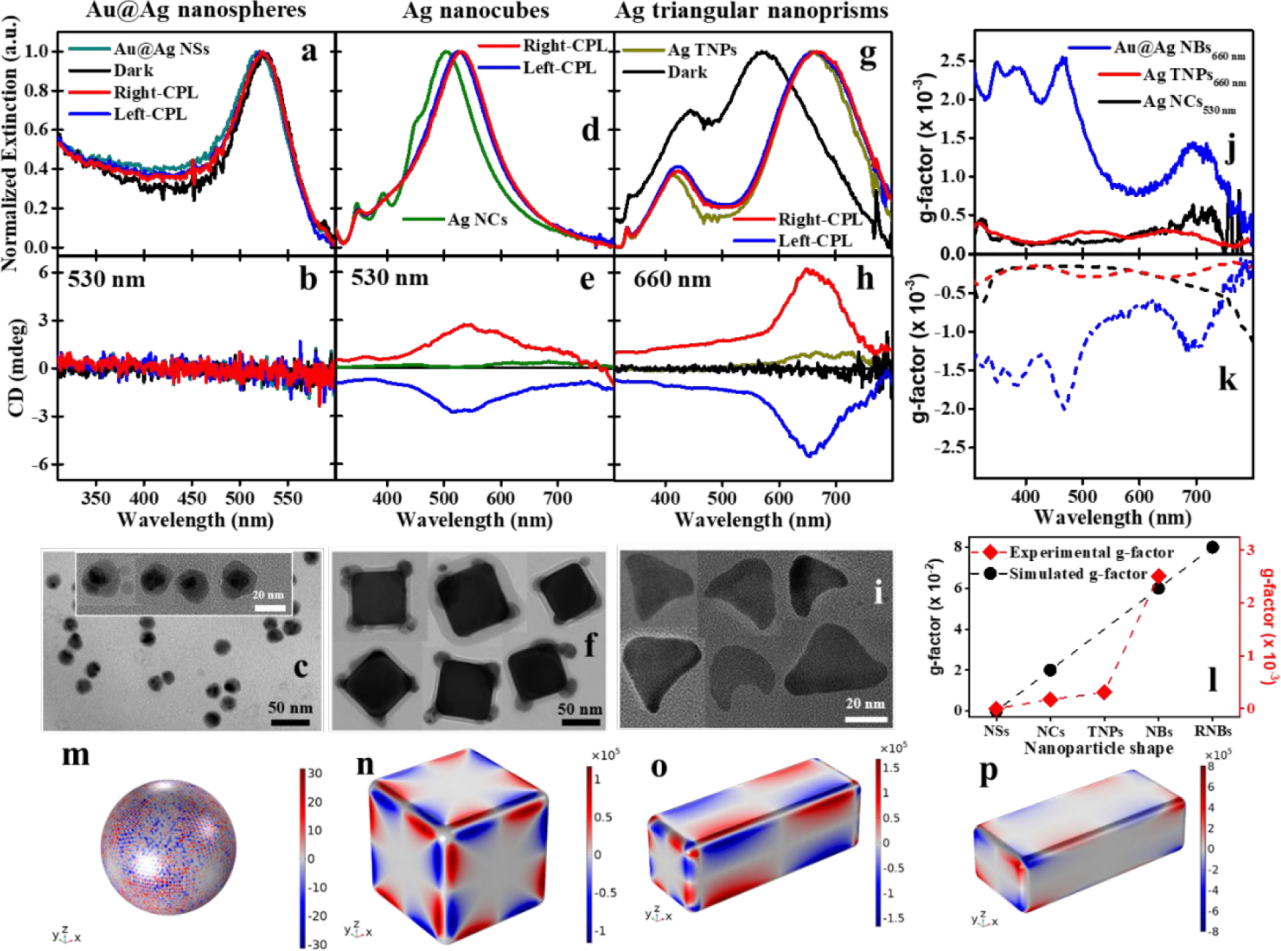
Results for NPs of different shapes. (a) Extinction spectra of the Au@Ag NSs, as prepared, after dark GRR, and after GRR with right and left-CPL illumination at 530 nm. (b) CD spectra of the Au@Ag NSs of the same samples. (c) TEM images of NSs after the GRR under right-CPL illumination. (d) Extinction spectra of the Ag NCs before and after GRR under left- and right-CPL illumination at 530 nm. (e) The corresponding CD spectra for the NCs before and after GRR under CPL illumination. (f) TEM images of the Ag NCs after GRR under right-CPL illumination. (g) Extinction spectra of Ag TNPs, as prepared, and after GRR in the dark and GRR under left- and right-CPL illumination at 660 nm. (h) CD spectra for the same Ag TNP samples. (i) TEM images of the Au@Ag TNPs after GRR under right-CPL illumination. The Au:Ag atomic ratio was 1:100 in all of the aforementioned GRR cases. Inset: TEM images. (j,k) Dissymmetry factor (*g*-factor) spectra of the Au@Ag NBs, Ag TNPs, and Ag NCs after GRR under right- and left-CPL illumination. (l) Comparing the simulated and experimental peak *g*-factor values of the different NPs shapes after GRR under right- CPL illumination, including more asymmetric NBs, called RNBs, with a rectangular cross-section. (m–p) The simulated surface mapping of relative HE generation rates for different shapes of NPs resonantly excited with CPL (adapted with permission from Figure 9 of ref, (25) copyright Wiley-VCH).

Movsesyan et al. estimated from simulations of HE induced metal deposition under CPL illumination in solution configuration that the magnitude of the resulting shape asymmetry and optical activity decreases as the illuminated original metal NPs become more symmetric.^25^ Consequently, compared to nanocubes (NCs) or nanospheres (NSs), elongated NBs are expected to have relatively stronger optical activity after GRR under CPL illumination, compared with more symmetric shapes as shown here experimentally:

We prepared a series of more symmetric silver-based nanostructures and examined their GRR behavior under CPL illumination: we prepared TNPs with an average edge length of 50 ± 10 nm, similar to the ones used by Sastry and coworkers,^13^ and core–shell Au@Ag NSs of ∼15 nm size (Figure S33). We also purchased silver NCs with an edge length of 75 nm (Nanocomposix). Figure 8a–i displays TEM images of the three nanostructures, after GRR + CPL illumination at the indicated (optimal) wavelengths with 1:100 Au:Ag atomic ratio, and the resulting CD spectra, compared to control samples of dark and LPL-illuminated GRR. Indeed, as the simulations predict, the silver TNPs and silver NCs produce weaker CD signals compared to the elongated NBs, and the Au@Ag NSs show zero CD signals at repeated experiments with varying illumination wavelengths and Au:Ag ratios. As in the case of the elongated NBs, the CPL illuminated NCs and TNPs exhibited CD spectra of a single polarity, which inverted with the change in handedness of the CPL.

The NBs exhibit a significantly higher *g*-factor than the other two nanocrystal types (see Figure 8j–l), in agreement with the theoretical predictions.^24,25^ The differences in the simulated localized field and consequent hot carrier distributions on excitation of the different NP shapes with CPL from multiple directions can be seen in Figure 8m–p. It can be clearly seen that hot carrier excitation in NSs is very low, while the elongated NBs have stronger and more localized plasmonic hot-spots relative to the NCs. Another type of NBs, with rectangular cross-section. i.e., more asymmetric, have slightly higher local fields and simulated CD g-factor, compared with regular NBs.

### 2.6 Plasmon Induced GRR Mechanism

It therefore seems that the symmetry-breaking mechanism occurring by CPL-controlled GRR follows the same qualitative behavior as the CPL-induced plasmonic HE based model used for the gold deposition in the sense that both correspond to excited asymmetric plasmonic hot spots (Figure S34).^24,25^ The exact nature of the plasmonic hot-spot-driven mechanism of GRR control should now be discussed: It has been observed in several experiments that aqueous colloids of silver NPs undergo enhanced etching (oxidation) under illumination at their plasmon resonance wavelength.^32,33^ Hence, it is plausible that silver oxidation through hot-hole generation at plasmonic hot-spots would be the driving force of GRR control. This can be compensated by excess electron scavenging of adsorbed AuCl_4_^–^ ions at the NP’s surface, with consequent gold deposition, which seems to occur near the hot spots. As observed in Figure 5e, Ag^+^ redeposition through electron scavenging seems to occur in significant proportion at the edges, near the hot spots.

Photothermal effects are unlikely to contribute to the asymmetric GRR as the light intensity used in all the experiments is relatively weak (order of ∼100 to 200 mW/cm^2^). In order to obtain significant photothermal effects in colloidal solutions higher peak intensity of pulsed laser irradiation is required.^34^

Inductively coupled plasma mass spectrometry (ICP-MS) was utilized to analyze the concentration of free gold and silver ions in solution after completion of the GRR under CPL-illumination and compare it with dark conditions. About 52%, 65%, 64%, 65%, and 61% of the AuCl_4_^–^ ions added for the GRR were consumed for dark, and 530, 565, 660, and 730 nm CPL-illumination, respectively (Table S2). This implies that more AuCl_4_^–^ ions are reduced on the surface of NBs when illumination is present. This agrees with the findings of Sastry and coworkers.^13^

For the GRR in the dark, the molar ratio of consumed gold ions to released silver ions was 1:2.85, which is close to the theoretically expected ratio of 1:3. However, this ratio increases under photoexcitation to 1:2.40, 1:2.27, 1:2.20, and 1:2.54 under 530, 565, 660, and 730 nm illumination, respectively, with the highest Au:Ag ratio occurring with 660 nm illumination. This implies that there are additional reaction pathways involved under plasmonic-controlled GRR, where the charge balance requires additional redox processes to occur. This should also explain the plasmon-assisted increased metal deposition. For example, chloride ions, released from the AuCl_4_^–^ ions as GRR progresses may act as hole scavengers,^35,36^ and would thus help maintaining the charge balance against HE injection to the metal ions at the hot-spots. Plasmon induced silver dissolution and redeposition has been observed in the shape transformation (ripening) of Ag NPs during light-mediated growth of Ag nanostructures.^11,37^

The silver ion concentration found in the solutions after the GRR is on the order of 7 μM (Table S2). The added AuCl_4_^–^ ions are typically of the order of ∼5 μM, hence the chloride ion concentration released during the GRR should be of the order of 20 μM. The solubility product of AgCl is ∼1.6 × 10^–10^, hence our chloride and silver ion concentrations are slightly below the solubility limit and not supposed to cause AgCl precipitation. However, we believe that there might be a substantial concentration of chloride ions at the surface of the NBs, in the form of AgCl, as noted above. We checked for the presence of chlorine in the EDS-STEM scans and detected a low concentration of chloride ions (as well as oxygen) at the surface of the illuminated NBs post GRR. The Cl/Ag atomic ratio was barely measurable, on the order of 0.1% in the NB shell.

To make sure that surface deposited AgCl or Ag_2_O is not directly responsible for the observed CD, we performed a control experiment in which sodium borohydride was added to the NBs solution post CPL-illuminated GRR and observed no change in CD and absorption spectra. Borohydride is able to reduce both AgCl and Ag_2_O to metallic silver; hence, the lack of spectral changes indicates that those species are not related to the observed CD.

### 2.7 Understanding the CD Spectra

A surprising aspect of the obtained CD spectra is that they are all unipolar, while previous studies of helical–chiral metal nanostructures typically produce bisignate lineshapes.^3,4,10,38^ Nevertheless, the previous simulations of orientationally averaged CPL-induced metal growth in similar gold nanostructures resulted in very small unipolar CD spectra (Figure S35).^24^ A simplistic model of asymmetric NBs shape with one to four spherical bulbs attached to the corners of the NBs (Figure S36) resulted in bisignate CD spectra, which was similar to the spectra calculated for the real individual Left-CPL particles (of opposite handedness, Figure 4d). The longitudinal plasmon resonance of the cuboid + bulb model shows a monosignate CD peak with polarity insensitive to the geometric details, and another CD line at ∼450 nm follows the same polarity as the longitudinal one, while the main transverse plasmon resonance changes CD line shape with geometric model (Figure S36). Averaging over many such shape variants might diminish part of it close to zero. This could explain why the asymmetric shape variety causes the averaging out of inverted polarity peaks in the CD spectrum of an ensemble of such symmetry-broken NBs.

### 2.8 CPL Illumination of NBs Attached to a Substrate

The symmetry breaking due to asymmetric plasmon excitation is supposed to be significantly stronger for a directional illumination case,^17,25^ where the NBs are immobilized on a surface. Figure 1b displays several SEM images of individual NBs deposited on a silicon substrate and going through the GRR under CPL illumination at 660 nm with light incidence perpendicular to the substrate. Strongly asymmetric 2D chiral shapes are clearly seen for 660 and 565 nm wavelengths (see also Figure S37), and to a lesser degree for illumination with 530 and 730 nm wavelengths, with their handedness correlated to the CPL handedness.

## 3 Conclusion

This work demonstrates that despite orientational averaging of asymmetric plasmon excitations in colloidal plasmonic NBs, there can be substantial chiral asymmetry formed in the shape of the NBs through plasmon-induced photochemical effects. In this study, fine-tuning of GRR under CPL illumination led to the appearance of significant shape and chiroptical dissymmetry, and it is conceivable that further optimizations could lead to even stronger effects. Further work on the GRR under directional CPL illumination of dense arrays of NBs could produce highly optically active metasurfaces. In addition, the clear correspondence between the asymmetric metal deposition and asymmetric plasmonic hot-spots could lead to a better understanding of plasmonic excitation induced redox processes with future additional experimental work on this mechanistic aspect.

## 4 Methods

The reactions with the Au@Ag NBs were carried out both in aqueous colloidal dispersion and under surface-confined conditions to examine the impact of CPL-induced plasmonic excitation on the GRR. Furthermore, the same methodology was also applied to three other symmetric geometrical shapes of silver NPs: TNPs, NCs, and Au@Ag NSs for CPL-directed GRR and compared with the corresponding control reactions in the dark.

### 4.1 Synthesis of Au@Ag NBs

In the first step, Au NRs were prepared following a previously published protocol with minor modifications:^39^ colloidal Au seed particles of ∼3 nm were produced by mixing 5.0 mL of 0.2 M cetyltrimethylammonium bromide (CTAB) aqueous solution with 5.0 mL of 0.5 mM HAuCl_4_ solution. Then 1.0 mL of ice-cold, freshly produced 6.0 mM NaBH_4_ solution was added to the CTAB/Au(III) solution while being magnetically stirred (1200 rpm). After stirring the seed solution for 60 s, it was left undisturbed for 30 min.

Afterward, 0.154 M of 125 mL of CTAB and 0.032 M of 125 mL of sodium oleate (NaOL) were dissolved together at 60 °C as the basis for the Au NR growth solution. After the solution has cooled to 30 °C, 18 mL of 4.0 mM silver nitrate (AgNO_3_) solution were added. After additional 15 min at 30 °C, 250 mL of 1.0 mM HAuCl_4_ were added to the mixture. After ∼90 min of magnetic stirring at 700 rpm, the solution turned colorless. At that point, 1.5 mL of hydrochloric acid (HCl; 37 wt % in water, 12.1 M) were added to the mixture. 1.25 mL of 64 mM ascorbic acid (AA) were added after additional 15 min of slow magnetic stirring at 400 rpm. Finally, 0.8 mL of the seed solution were added into the growth solution. The mixture was rapidly agitated for an additional 30 s and then left undisturbed at 30 °C for 12 h. The resultant Au NRs were purified by centrifugation at 7000 rpm for 20 min, removing the supernatant, and then redispersed in 30 mL of a 20 mM CTAB solution.

For the coating of silver to form Au@Ag NBs: 100 μL of gold NR solution were centrifuged at 6000 rpm for 20 min and the NR precipitate was dissolved in 2 mL of a 80 mM cetyltrimethylammonium chloride (CTAC) solution. This centrifugation procedure was repeated twice, and finally, the gold NRs were redispersed in 100 μL of 80 mM CTAC solution. In a 20 mL glass vial, 10 mL CTAC solution (80 mM) was heated to 70 °C and 100 μL of the NR-CTAC solution were added with gentle stirring. After 2 min, 0.5 mL of 100 mM AA solution were added. After mixing this solution for 5 min, 0.06–2.0 mL of 0.5 M sodium hydroxide solution were added while vigorously stirring, to obtain pH ≈ 3. This was followed by the addition of 75 to 750 μL of 10 mM silver nitrate (depending on the desired silver thickness). The reaction mixture was kept at 70 °C for 3 h. The resultant Au@Ag NBs were purified by centrifugation at 7000 rpm for 20 min, removing the supernatant, and dispersing the NBs in ultrapure water. The structural details of the NBs with different silver thicknesses are listed in Table S1.

### 4.2 Synthesis of Triangular Ag TNPs

A seed-mediated procedure was used to prepare silver TNPs.^13^ In the first step, Ag seeds were prepared by mixing aqueous trisodium citrate (TSC; 5 mL, 2.5 mM), aqueous poly(sodium styrenesulfonate) (1000 kDa, 0.25 mL, 500 mg/L), and aqueous NaBH_4_ (0.3 mL, 10 mM, freshly prepared) followed by addition of aqueous AgNO_3_ solution (5 mL, 0.5 mM) at a rate of 2 mL/min while stirring. The seed particles were allowed to grow for 20 min. Subsequently, Ag TNPs were grown by combining 5 mL of ultrapure water, 75 μL of aqueous AA solution (10 mM), and 60 μL of the Ag seed solution and then adding 1.5 mL of aqueous AgNO_3_ (0.5 mM) at a rate of 1 mL/min. The synthesis was stopped by a quick addition of 0.5 mL of aqueous TSC, 25 mM.

### 4.3 Commercial Ag NCs

The 75 nm silver NCs were bought from NanoComposix (San Diego, CA). These NCs have PVP-stabilized surfaces and are supplied at a concentration of 1 mg/mL in ethanol. The Ag NCs were diluted to the desired concentration in water.

### 4.4 Synthesis of Au@ag NSs

In order to synthesize core–shell Au@Ag NSs, the Turkevich method was first used to prepare spherical Au cores:^40^ 50 mL of 0.25 mM HAuCl_4_ were heated to 100 °C in a round-bottom flask with vigorous stirring. Then, 1176 μL of 34 mM TSC was quickly added into the flask. The reaction mixture was allowed to cool to room temperature after 15 min of stirring.

Then, to grow the Ag shell, 2 mL of CTAB (100 mM) was gently stirred in a glass vial with 2 mL of the previously made Au NPs at 40 °C. Afterward, 16 μL of AgNO_3_ (10 mM), 500 μL of AA (0.1 M), and 200 μL of NaOH (0.5 M) were added. Following each step of addition, the reaction was gently mixed for 2 min. It took five more minutes of mixing to complete the silver deposition reaction. To avoid CTAB crystallization, the NPs were stored at 40 °C for the GRR.

### 4.5 GRR Under Illumination in Colloidal Solution

0.8–1.0 mL aliquots of NPs (NBs, TNPs, NCs or NSs) were placed in 12.5 mm diameter cylindrical glass vials and subjected to continuous-wave polarized LED illumination with either left- and right-CPL, or LPL, using broad band linear wire-grid polarizer (Thorlabs WP25M-VIS) and achromatic quarter waveplate (Thorlabs AQWP10M–580), with output intensities in the range ∼100–200 mW/cm^2^. The illumination wavelengths of the LEDs used were 530 nm (Thorlabs model M530L4), 660 nm (Thorlabs M660L4), 565 nm (Thorlabs M565L3), and 730 nm (Thorlabs M730L5). The LED light was slightly focused to a square spot of ∼1 cm^2^ with a collimator + a focusing lens illuminating the solution through the side wall of the vial, overlapping about half of the solution volume. A quick addition of HAuCl_4_ (0.8–1 mL) solution, with an Au:Ag molar ratio (added gold relative to the amount of silver deposited in the coating) of 1:200 up to 1:10 was done after 1 min under LED illumination. The GRR was carried out for 5 min under illumination and magnetic stirring. A control reaction was performed under identical conditions but in complete darkness.

### 4.6 GRR Under Illumination for NBs Deposited on a Substrate

For substrate-confined GRR, a cleaned highly doped Si wafer was dipped into the solution mixture of 200 μL of Au@Ag NBs and 2 μL of sodium dodecyl sulfate (5 mM) for 12 h. After being washed twice with methanol and distilled water, the Si substrate was stored under vacuum to avoid silver shell oxidation in humid air. Afterward, the GRR was performed by exposing the substrate to the LPL/CPL illumination (530, 565, 660, and 730 nm) for 1 min followed by addition of 1 mL of 1 μM HAuCl_4_ solution on the Si substrate (in a small Petri dish) with mild magnetic stirring at 100 rpm on the side of the substrate. The illuminated reaction was performed for 5 min. At the end of the reaction, the HAuCl_4_ solution was extracted using a dropper, and the Si substrate was washed twice with methanol and distilled water and stored under vacuum for SEM imaging.

### 4.7 Characterization

#### 4.7.1 Spectroscopy

UV–visible extinction spectra were measured by using a fiber-optic-based StellarNet BlackComet spectrometer. CD measurements were performed in a Chirascan CD spectrometer (Applied Photophysics, UK) at a 280–800 nm wavelength range with a 3 nm bandwidth.

#### 4.7.2 Electron Microscopy

TEM images were acquired using a Thermo Fisher Scientific Talos F200i (S)TEM (100 keV). SEM imaging was done in a Zeiss Gemini SEM 300. Sample preparation for TEM analysis was done by drop casting the NP solution on a carbon-coated Cu TEM grid, washing twice with methanol and distilled water, and drying in air. NB geometry statistics was performed on TEM images by measuring over 50 NBs using ImageJ software. STEM-EDS chemical mapping was conducted in a Spectra 200 TEM (Thermo Fisher), employing an acceleration voltage of 200 keV, a camera length of 98 mm, and a probe current of ∼180 pA. Images were recorded with a HAADF detector (56–200 mrad collection angle) and a BF/DF Panther segmented detector (BF, 23 mrad collection angle). Data analysis was performed by using Velox 3.11 (Thermo Fisher Scientific).

#### 4.7.3 ICP-MS

The NB solutions post GRR under both dark and CPL-illumination conditions were centrifuged at 15,000 rpm for 4 min to precipitate out the NPs, following which the supernatants were collected for evaluation. The concentrations of Ag^+^ and Au^3+^ ions in these solutions were determined by inductively coupled plasma mass spectrometry (Agilent model 7800) to monitor the AuCl_4_^–^ consumption and Ag^+^ dissolution yields. This was accomplished by heating a mixture of 0.5 mL of supernatants with 1.5 mL of 32% hydrochloric acid and 0.5 mL of 67% nitric acid for 1 h at 75 °C. The mixture was further diluted with distilled water to a final volume of 50 mL, and then injected into the ICP-MS instrument.

#### 4.7.4 3D Electron Tomography Reconstruction

TEM specimens for 3D reconstruction were prepared by drop-casting Au@Ag NB dispersions on a holey carbon coated Cu TEM grid (200 mesh). 3D tomography data acquisition was performed using Talos F200X G2 TEM with an accelerating voltage of 200 keV and the software SerialEM.^41^ STEM-HAADF imaging in the dark field mode was selected to perform the 3D reconstruction of the Au@Ag NBs to clearly reveal the contrast of Au NRs and the Ag shell. Electron dose rate was carefully controlled to avoid beam damage under a long acquisition time. The tilting range was ±60° with 2° interval. The CCD spikes in the images were removed first, and then the images were aligned by the cross-correlation method. The processed image data set was further analyzed using the IMOD software,^42^ and reconstructed through a simultaneous iterations reconstruction technique (SIRT) with 10 iterations.

### 4.8 Geometric Chirality of the NBs and Its Measures

#### 4.8.1 Orienting the Object to Obtain Its Mirrored Mesh (Opposite Enantiomer)

The 3D structure of NB was studied using a Monte Carlo approach. The 3D body is represented by a dense cloud of points, sampled with *N* = 5 × 10^6^ points in the minimal parallelogram containing the 3D mesh, and represented by the function ρ (*x,y,z*):=1 inside the NB and zero outside. The origin of coordinates is set at the center of mass of the NB, and its first two principal axes are aligned with the Z and Y Cartesian axes.

#### 4.8.2 To Determine the Presence of Chirality We Introduced the Following Measures

(1) The 2D chiral parameter:
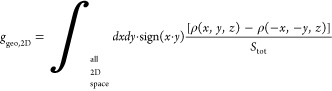


This parameter was calculated for various 2D cross sections along the *z*-axis of the 3D reconstruction for particular z values.

(2) The 3D chirality parameter:
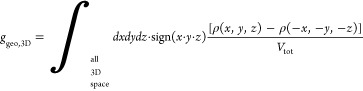


*g*_*geo*_,_3*D*_ is the normalized chirality measure averaged over all orientations of the object.

### 4.9 Electromagnetic Simulations

The computation of the chiro-optical responses of the NPs was performed with the COMSOL Multiphysics software. Each NB is composed of a gold core and a silver shell, while the surrounding medium is water. To calculate the extinction cross sections and their (extinction) CD spectra, we computed the absorption and scattering cross sections. The absorption cross section is given by
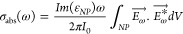
where Im (ε_NP_) is the imaginary part of the local dielectric function of the NP, *I*_0_ is the incident flux, and is the electric field generated inside the NP. Finally, the extinction cross section should be written as the sum of the absorption and scattering cross sections. The CD response of each NP is the average of six electromagnetic configurations when the incident electromagnetic field approaches the NB from three perpendicular directions and may have two polarization states.

## Data Availability

The data that support the findings of this study are available from the corresponding author upon reasonable request.

## Supporting Information Available

The size of the NBs prepared at different conditions, and additional CD and extinction spectra of different samples under different GRR parameters (Au:Ag ratios, illumination wavelength and intensity); electromagnetic simulation results for the modeled NBs before and after GRR as well as other shapes, in the form of extinction and CD spectra, electric field, electric charge, and pointing vector magnitude surface distribution maps; additional electron tomography data and shape chirality analysis and additional SEM/TEM images and Au/Ag distribution maps of the NBs; table of results of ICP-MS analysis. A previous version of this manuscript has been deposited on a preprint server: Monika Ghalawat, Daniel Feferman, Lucas Besteiro, Wanting He, Artur Movsesyan, Jesus Valdez, Audrey Moores, Zhiming Wang, Dongling Ma, Alexander Govorov, and Gil Markovich; “Chiral Symmetry Breaking in Colloidal Metal Nanoparticle Solutions by Circularly Polarized Light”; 2024, ChemRxiv (PDF)
