# Supplementary material for: Chiral Symmetry Breaking in Colloidal Metal Nanoparticle Solutions by Circularly Polarized Light
Source: ACS Nano. 2024 Oct 5;18(41):28279–91. doi: 10.1021/acsnano.4c09349 (PMC11483945; doi:10.1021/acsnano.4c09349)
Supplement: Supplementary file 1 — nn4c09349_si_001.pdf [file nn4c09349_si_001.pdf]

Supporting Information

Chiral Symmetry Breaking in Colloidal Metal Nanoparticle Solutions by Circularly Polarized Light

Monika Ghalawat[1], Daniel Feferman[1], Lucas V. Besteiro[2], Wanting He[3], Artur Movsesyan[4,5], Alina Muravitskaya[5], Jesus Valdez[6], Audrey Moores[6,7], Zhiming Wang[5], Dongling Ma[3], Alexander O. Govorov[4*], Gil Markovich[1*]

[1] School of Chemistry, Tel Aviv University, Tel Aviv 6997801, Israel.

[2] CINBIO, University of Vigo, 36310 Vigo, Spain.

[3] Énergie Matériauxet Télécommunications, Institut National de la Recherche Scientifique (INRS), 1650 Bd Lionel-Boulet, Varennes, QC J3X 1P7, Canada.

[4] Department of Physics and Astronomy and Nanoscale and Quantum Phenomena Institute, Ohio University, Athens, Ohio 45701, USA.

[5] Institute of Fundamental and Frontier Sciences, University of Electronic Science and Technology of China, Chengdu 610054, China

[6] Facility for Electron Microscopy Research (FEMR), McGill University, 801 Sherbrooke Street West, Montréal, QC, H3A 0B8, Canada

[7] Centre in Green Chemistry and Catalysis, Department of Chemistry, McGill University, 801 Sherbrooke Street West, Montréal, QC, H3A 0B8, Canada

(* Email addresses: gilmar@post.tau.ac.il, govorov@ohio.edu)

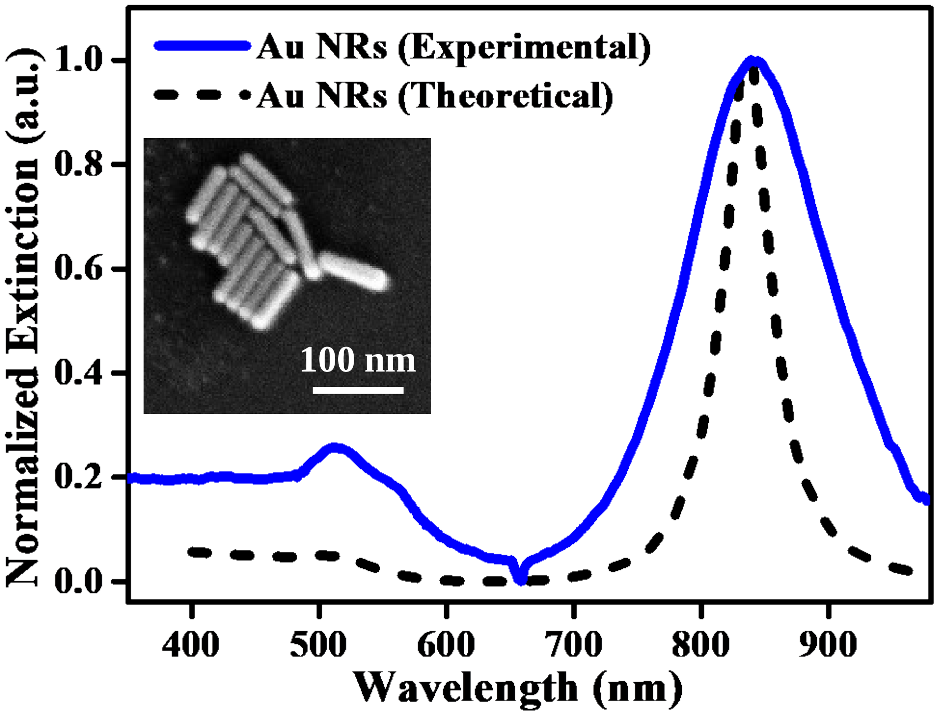

Fig. S1: Experimental and simulated extinction spectra of the core Au NRs. Inset: SEM image of the Au NRs.

Table S1: Dimensions of Au@Ag NBs synthesized with different silver coating thicknesses. Listed in the columns are the volumes of added precursors and reagents for six samples, with increasing shell thickness. The average length and width of all NBs listed here were estimated from SEM images. For Sample 2, a more precise average size estimate of the NBs was done based on TEM images, and is reported in the main text.

**Sample no.**	**Au NRs**	**AA (100 mM)**	**NaOH (0.5 M)**	**$AgNO_3$ (10 mM)**	**Average length (nm)**	**Average width (nm)**
**Sample 1**	100 µL	0.5 mL	0.06 mL	75 µL	110 ± 5	39 ± 4
**Sample 2**	100 µL	0.5 mL	0.06 mL	100 µL	102 ± 5	40 ± 4
**Sample 3**	100 µL	0.5 mL	0.25 mL	150 µL	101 ± 5	49 ± 4
**Sample 4**	100 µL	0.5 mL	0.5 mL	250 µL	111 ± 5	56 ± 4
**Sample 5**	100 µL	0.5 mL	1.0 mL	500 µL	121 ± 5	83 ± 4
**Sample 6**	100 µL	0.5 mL	2.0 mL	750 µL	129 ± 5	91 ± 4

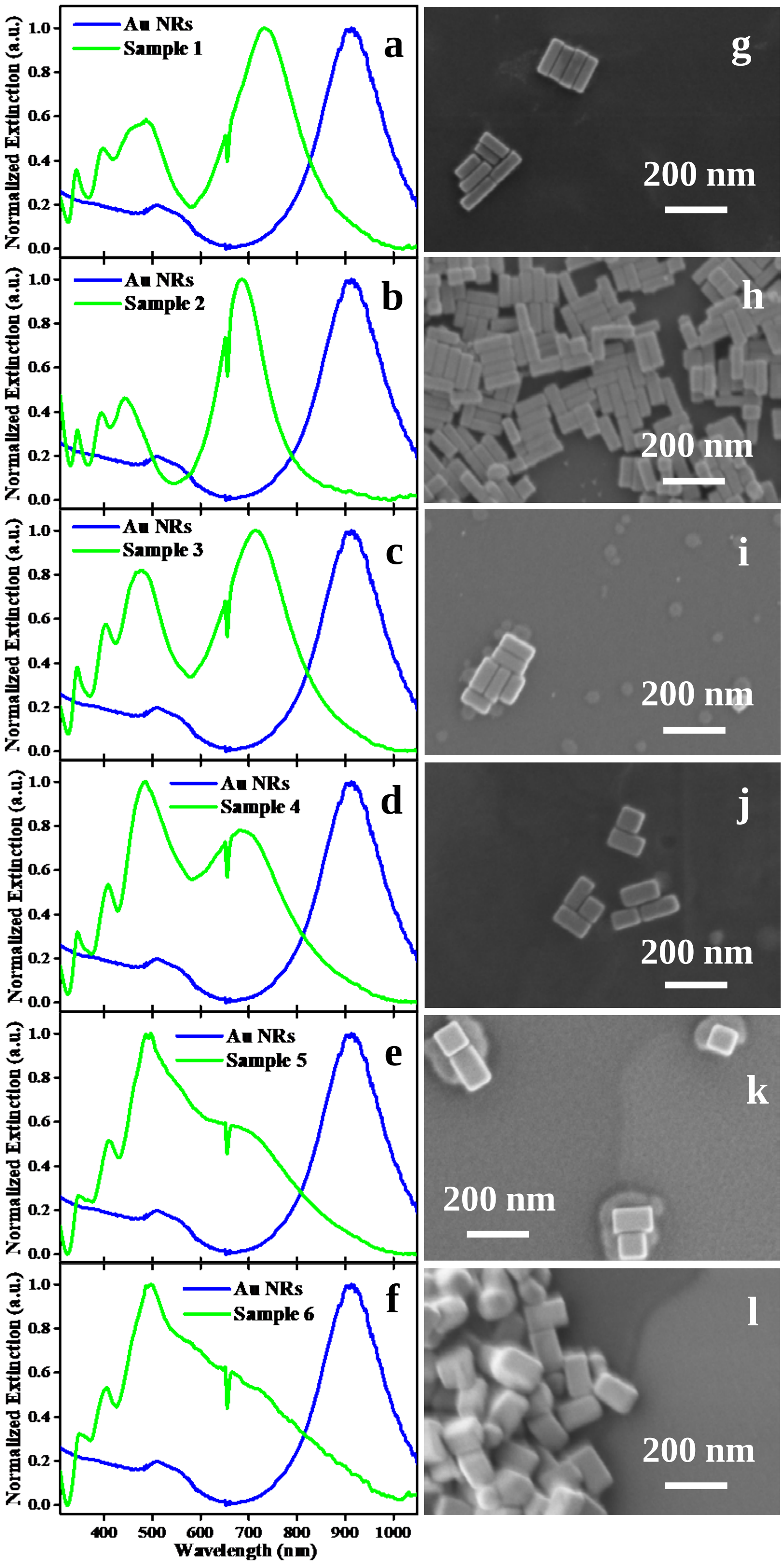

Fig. S2: (a-f) Normalized extinction spectra of the core Au NRs, and sample 1 to 6 Au@Ag NBs (with different silver coating thicknesses as listed in Supplementary Table 1). (g-l) Corresponding SEM images of the Au@Ag NBs.

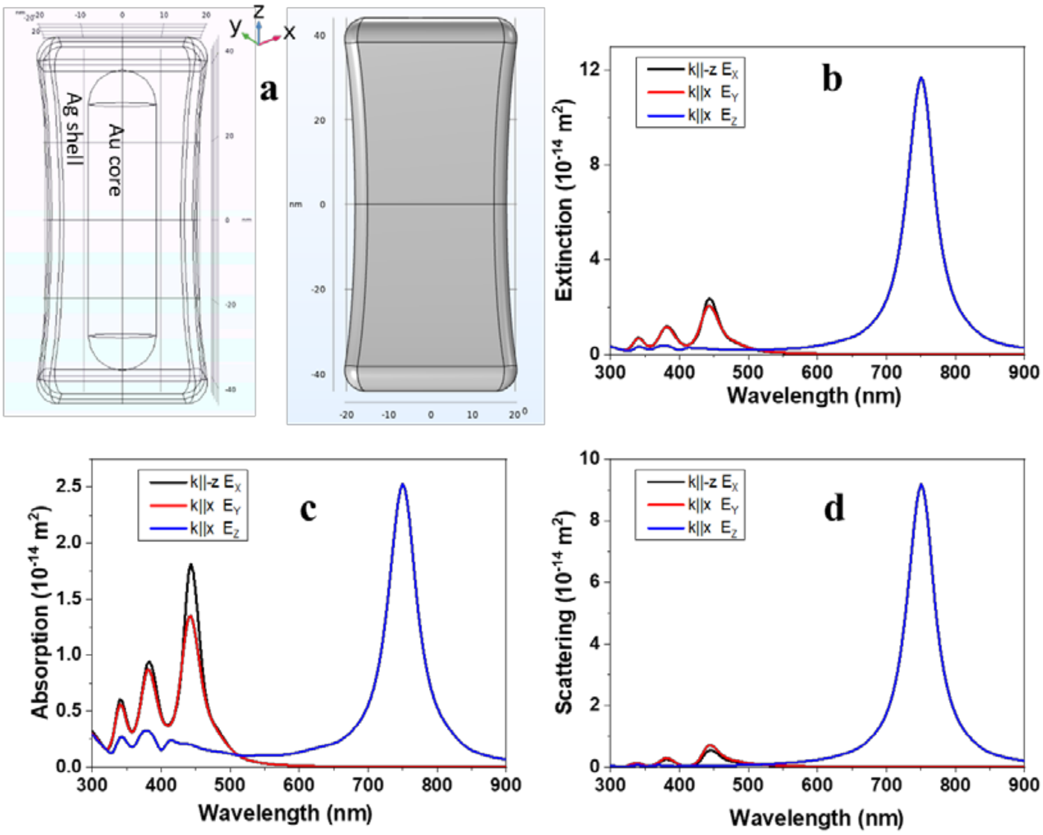

Fig. S3: Simulation results on the plasmon resonances of the Au@Ag NBs. The modelled shape is slightly concave to take account of the situation after the beginning of GRR, where some silver is dissolved from the centers of the elongated facets. The results are not sensitive to this concavity. (a) The geometrical model of the NBs. (b) The simulated extinction spectrum for different illumination directions (different k vectors) and different orientations of electric field (linear polarization). It can be seen that, as expected, the longitudinal resonance (at 750 nm) is excited only when light incidence is perpendicular to the z-axis. (c) The corresponding absorption and (d) scattering spectra components of the extinction spectra.

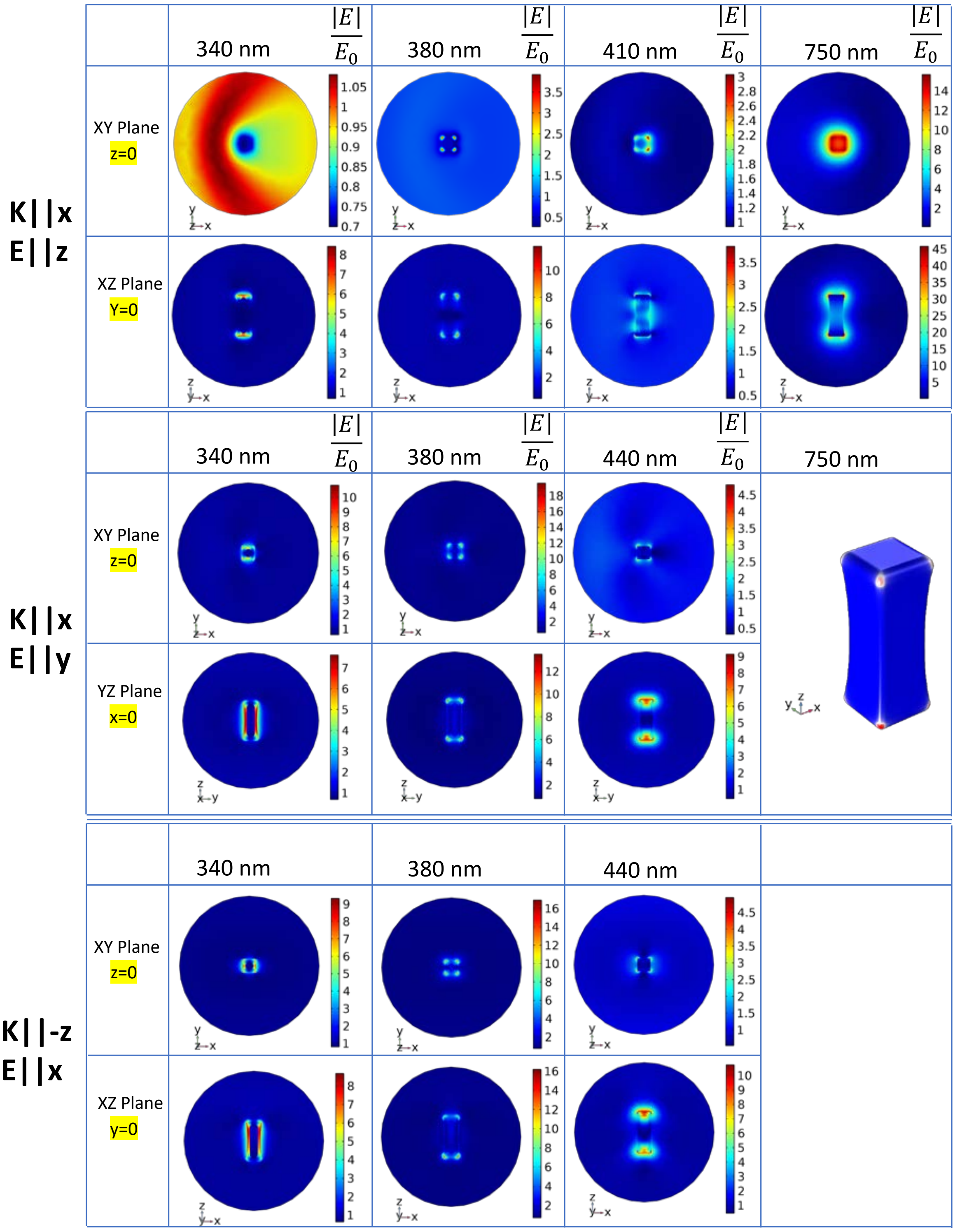

Fig. S4: Simulated electric field distributions for the different illumination incidence (k vector) and linear polarization (E vector) orientation, for the different resonance modes, with indicated wavelengths. Electric field values are normalized to the incident field amplitude. Note that there are some changes between the two sides of the NBs along the light propagation direction (k) due to interaction and losses during passage through the NBs, since the effective wavelength in the metal is much smaller than the vacuum wavelength.

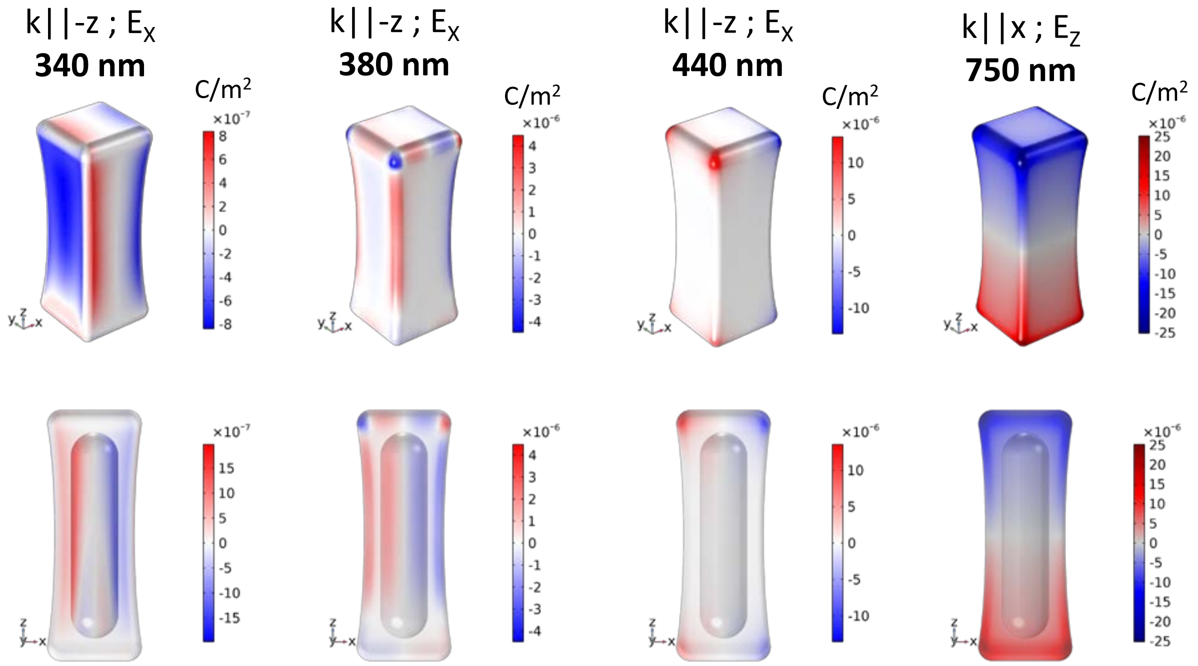

Fig. S5: Simulated surface charge distribution snapshots (imaginary component) at the four different plasmon resonances, for different light incidence and linear polarization orientations. In the bottom row, the silver shell was made partially transparent to show the charge distribution at the gold-silver interface. Note in particular the 340 nm resonance, which is called silver "slab" resonance, and is a dipole oscillation across the silver shell.

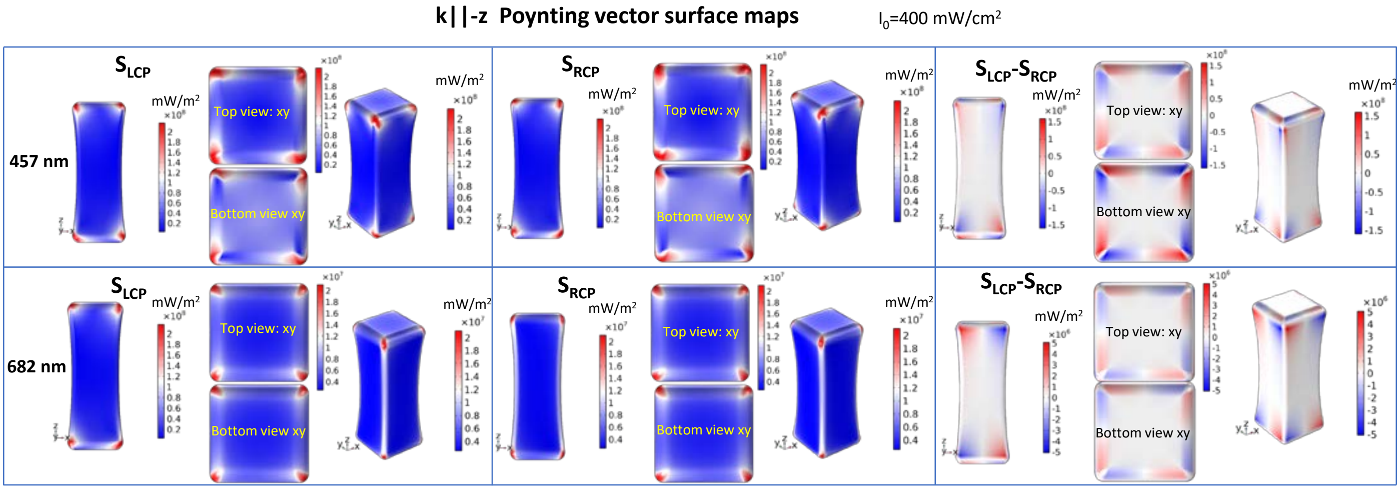

Fig. S6: Simulated pointing vector magnitude distribution for the two circular polarization illumination conditions (S_{LCP} and S_{RCP}), time averaged. Light incidence along the long axis of the NBs. These distributions represent the local energy flow with CPL excitations at the indicated wavelengths: 457 nm is very close to the transverse resonance mode (slightly red-shifted) and 682 nm is to the blue side of the longitudinal resonance. The two right panels are the calculated differences in Poynting vector between the RCP and LCP illuminated NBs, illustrating the asymmetries between the two CPL induced modes. It can be seen that the stronger absolute values of peaks and difference maps in S vector distribution are for the transverse mode. At both wavelengths, asymmetric distribution patterns can be observed around the apexes.

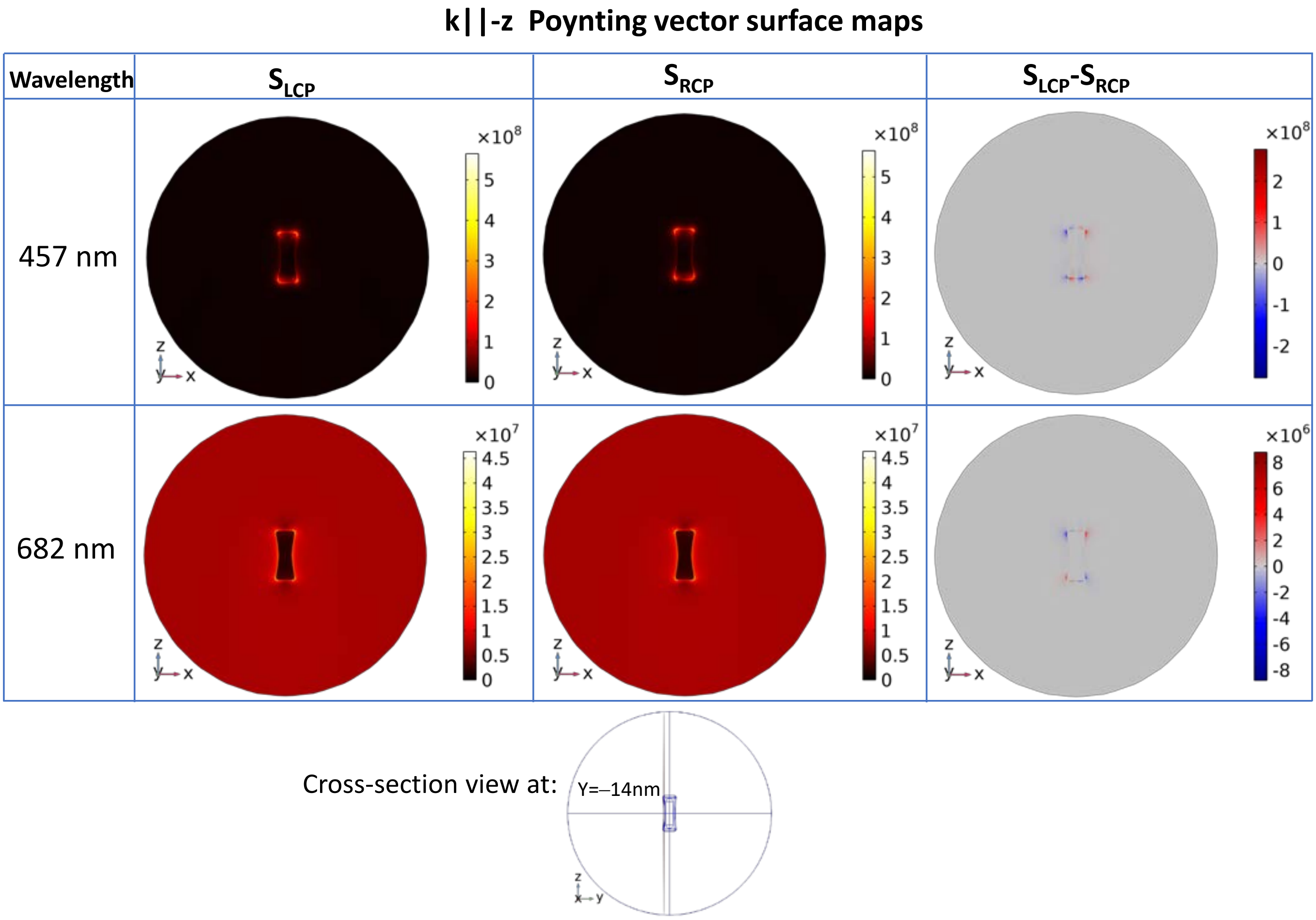

Fig. S7: Cross-sectional view of the simulated Poynting vector magnitude distribution for the two circular polarization illumination conditions (S_{LCP} and S_{RCP}), time averaged. Light incidence along the long axis of the NBs. The cross-section is at the X-Z plane within the silver shell. These distributions represent the local energy flow with CPL excitations at the indicated wavelengths: 457 nm is very close to the transverse resonance mode (slightly red-shifted) and 682 nm is to the blue side of the longitudinal resonance. The two right panels are the calculated differences in Poynting vector between the RCP and LCP illuminated NBs, illustrating the asymmetries between the two CPL induced modes.

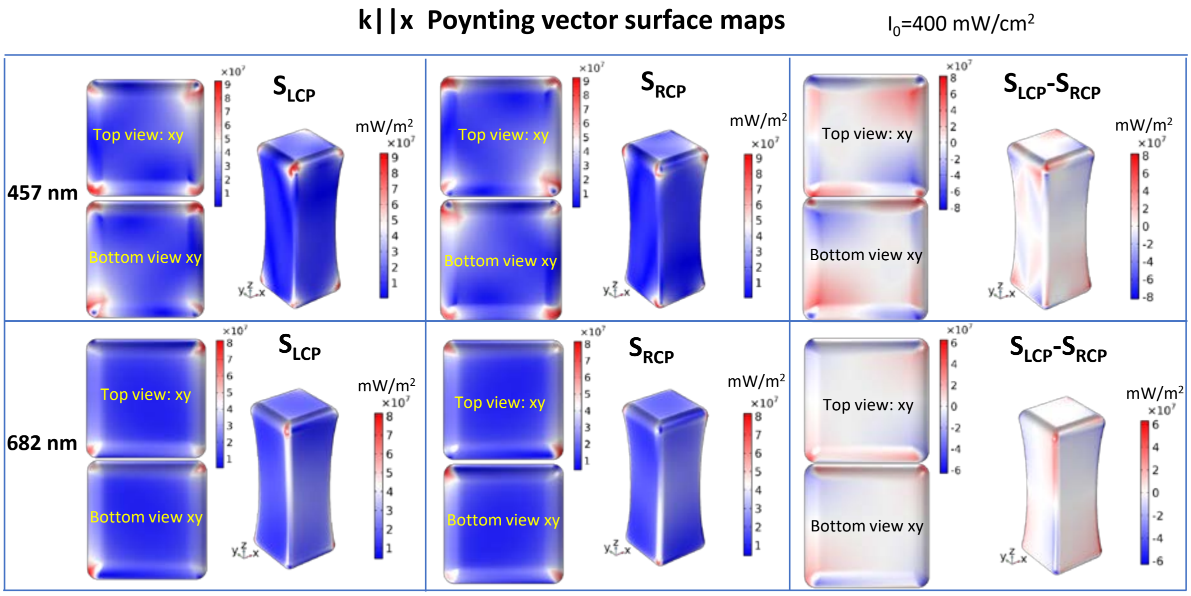

Fig. S8: Simulated pointing vector magnitude distribution for the two circular polarization illumination conditions (S_{LCP} and S_{RCP}), time averaged. Light incidence along the <u>short</u> axis of the NBs. These distributions represent the local energy flow with CPL excitations at the indicated wavelengths: 457 nm is very close to the transverse resonance mode (slightly red-shifted) and 682 nm is to the blue side of the longitudinal resonance. The two right panels are the calculated differences in Poynting vector between the RCP and LCP illuminated NBs, illustrating the asymmetries between the two CPL induced modes. In this case the absolute values of peaks and difference maps in S vector distribution are comparable for both wavelengths. At both wavelengths, asymmetric distribution patterns can be observed around the apexes, with stronger peak magnitude differences between adjacent apexes.

k||x Poynting vector surface maps

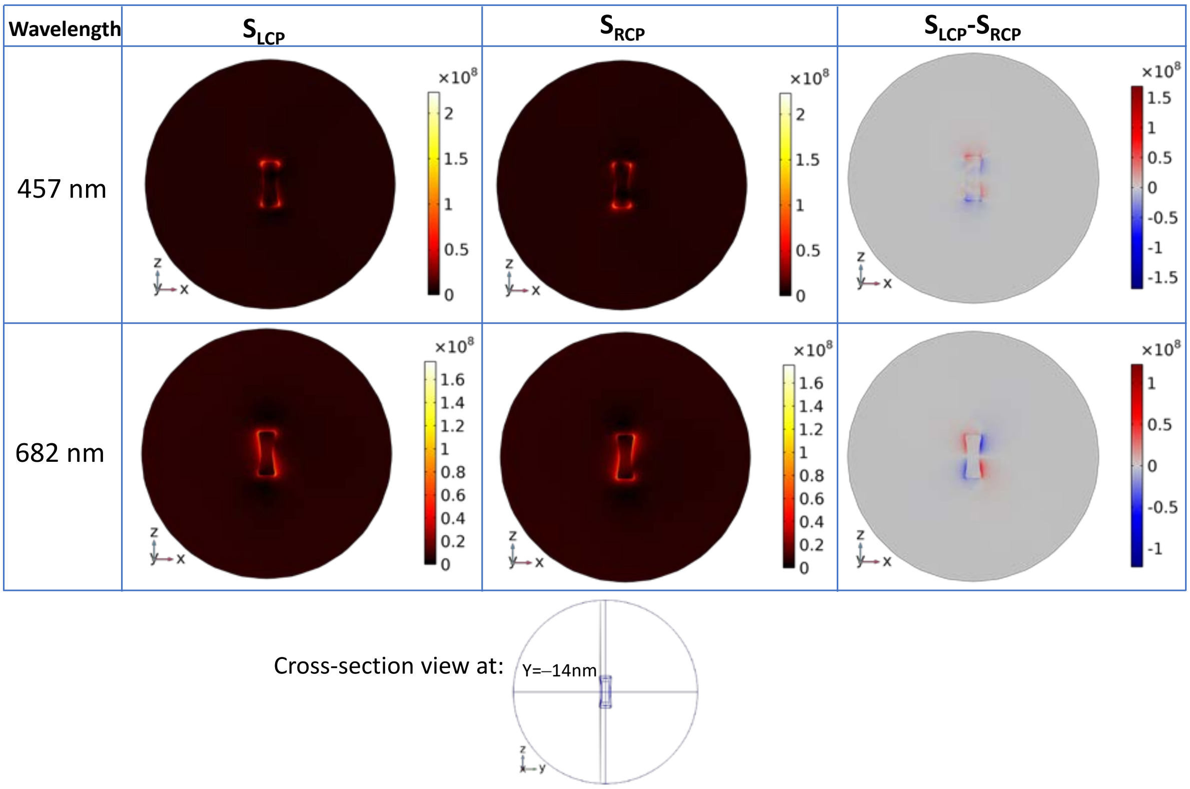

Fig. S9: Cross-sectional view of the simulated pointing vector magnitude distribution for the two circular polarization illumination conditions (S_{LCP} and S_{RCP}), time averaged. Light incidence along the <u>short</u> axis of the NBs. The cross-section is at the X-Z plane within the silver shell. These distributions represent the local energy flow with CPL excitations at the indicated wavelengths: 457 nm is very close to the transverse resonance mode (slightly red-shifted) and 682 nm is to the blue side of the longitudinal resonance. The two right panels are the calculated differences in Poynting vector between the RCP and LCP illuminated NBs, illustrating the asymmetries between the two CPL induced modes. In this case the absolute values of peaks and difference maps in S vector distribution are comparable for both wavelengths. At both wavelengths, asymmetric distribution patterns can be observed around the apexes, with stronger peak magnitude differences between adjacent apexes.

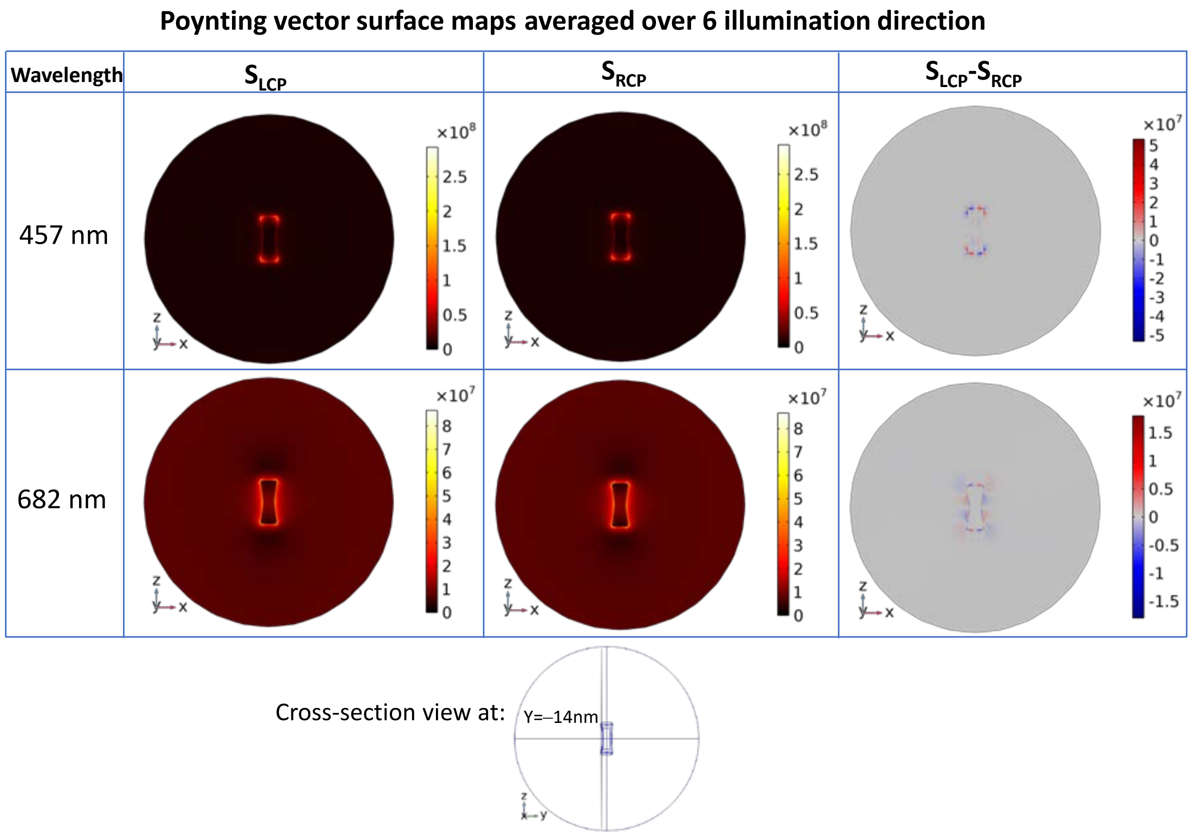

Fig. S10: Cross-sectional view of the simulated pointing vector magnitude distribution for the two circular polarization illumination conditions (S_{LCP} and S_{RCP}), time averaged and incidence direction averaged between the three axes, and for each axis, including both positive and negative directions to remove front and back surface asymmetry. The cross-section is at the X-Z plane within the silver shell. These distributions represent the local energy flow with CPL excitations at the indicated wavelengths: 457 nm is very close to the transverse resonance mode (slightly red-shifted) and 682 nm is to the blue side of the longitudinal resonance. The two right panels are the calculated differences in Poynting vector between the RCP and LCP illuminated NBs, illustrating the asymmetries between the two CPL induced modes. While the absolute values of peaks and difference maps in S vector distribution are larger for both the transverse resonance, the relative differences are comparable for both wavelengths. At both wavelengths, it is difficult to find asymmetric distribution patterns for the individual polarizations, but in the difference maps they are clearly observed.

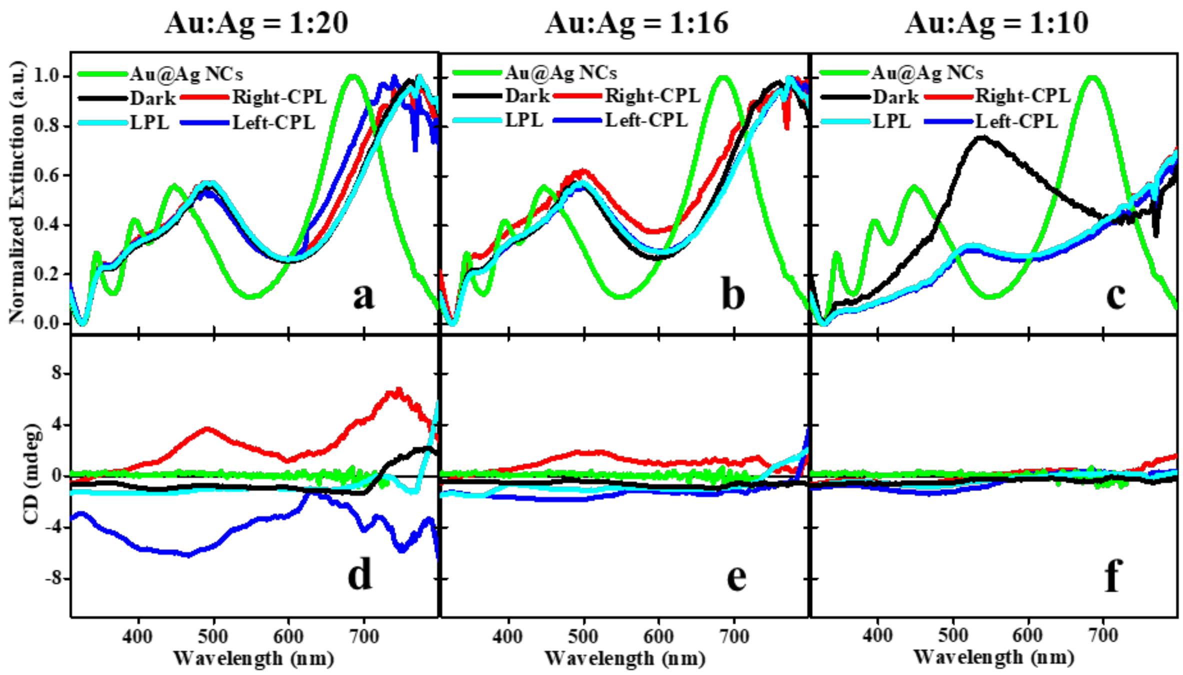

Fig. S11: (a,b,c) Normalized extinction spectra of the original Au@Ag NBs, NBs after GRR in the dark, after GRR under LPL illumination and after GRR under right- and left-CPL illumination at 660 nm (with the indicated Au:Ag atomic ratios). (d,e,f) Corresponding CD spectra of the same samples. All performed with NBs of sample 2.

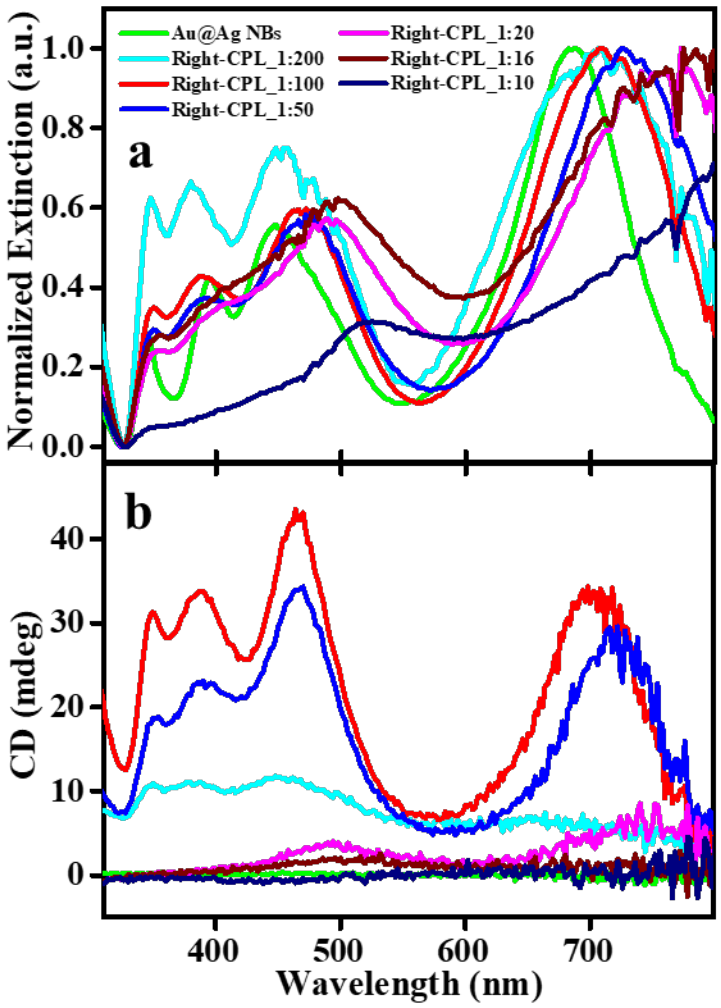

Fig. S12: (a) Normalized extinction spectra of the original Au@Ag NBs (Sample 2) and NBs after GRR under right-CPL illumination at 660 nm (with the indicated Au:Ag atomic ratios), (b) The corresponding CD spectra of the same samples.

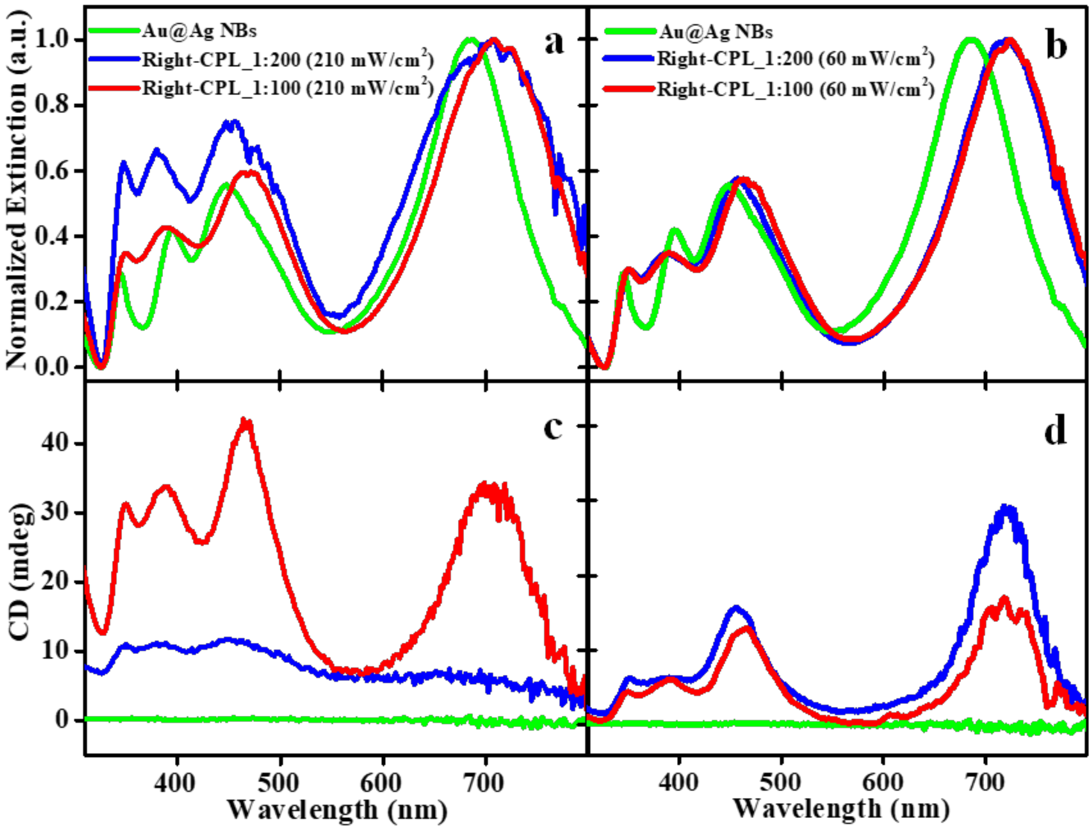

Fig. S13: Normalized extinction spectra of the original Au@Ag NBs and NBs after GRR under right-CPL 660 nm illumination with Au:Ag atomic ratio of 1:100 and 1:200 at (a) power ~210 mW/cm^2 and (b) power ~60 mW/cm^2. (c,d) The corresponding CD spectra of the same samples.

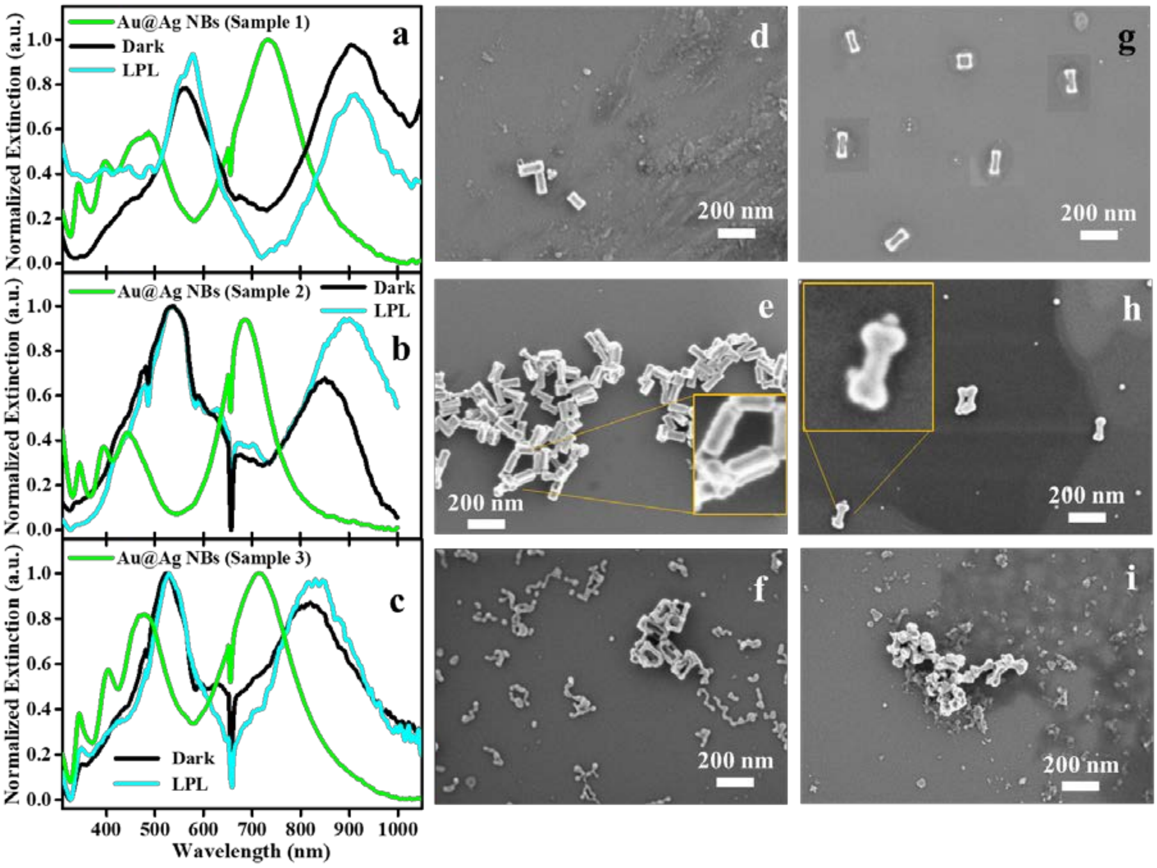

Fig. S14. (a,b,c) Normalized extinction spectra of the original Au@Ag NBs of samples 1-3, NBs after GRR in the dark, and after GRR under LPL illumination at 660 nm (with Au:Ag atomic ratio of 1:10). (d,e,f) The corresponding SEM images of the same samples after GRR in the dark. (g,h,i) The corresponding SEM images of the same samples after GRR under LPL illumination. In panel (g), several particle images from other frames were pasted to show more NPs.

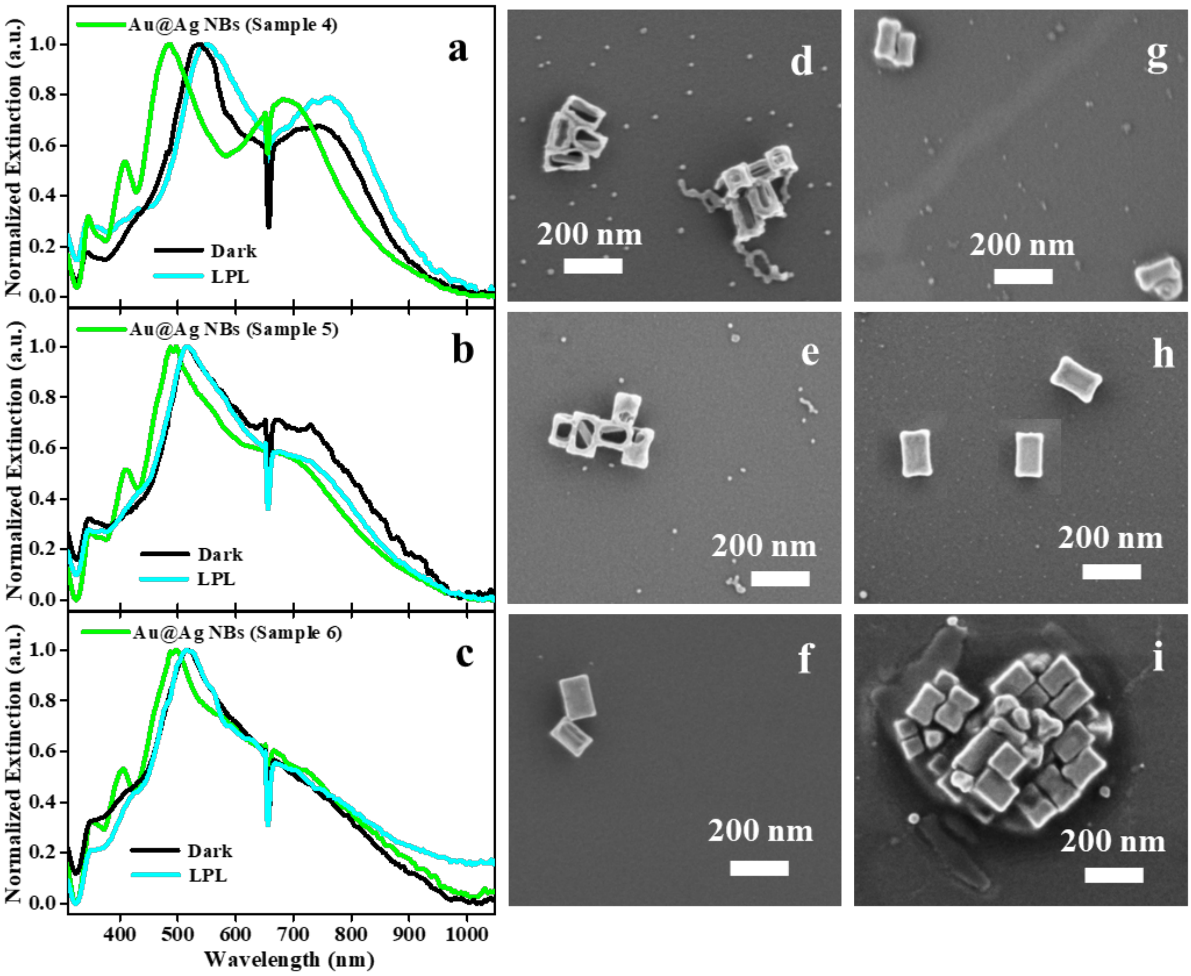

Fig. S15: (a,b,c) Normalized extinction spectra of the original Au@Ag NBs of samples 4-6, NBs after GRR in the dark, and after GRR under LPL illumination at 660 nm with Au:Ag atomic ratio of 1:10. (d,e,f) The corresponding SEM images of the same samples after GRR in the dark. (g,h,i) The corresponding SEM images of the same samples after GRR under LPL illumination. In panel (h), several particle images from other frames were pasted to show more NPs.

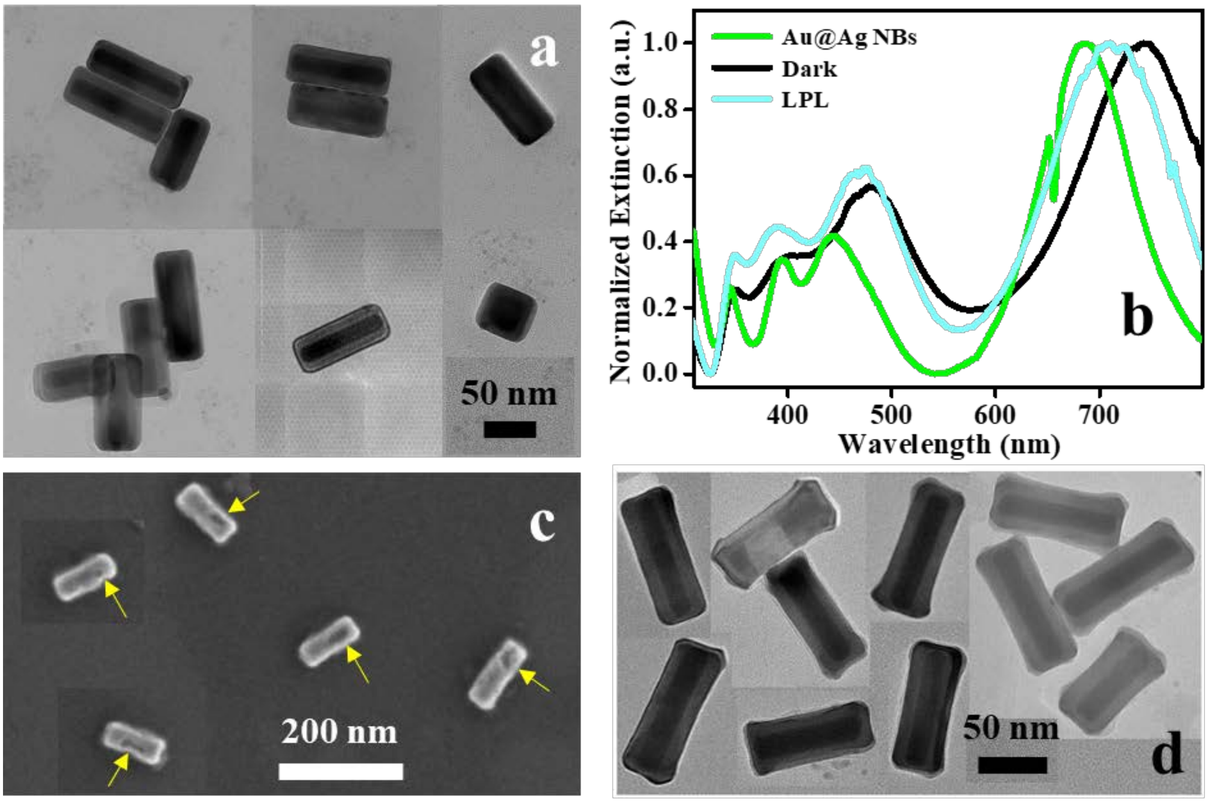

Fig. S16: (a) TEM images of the original Au@Ag NBs (Sample 2). (b) Normalized extinction spectra of the original Au@Ag NBs, after GRR in the dark and after GRR under LPL illumination at 660 nm (210 mW/cm^2) with Au:Ag atomic ratios of 1:100. (c) Corresponding SEM images of NBs after GRR in the dark, where the yellow arrows indicate a pinhole in each NB. (d) TEM images of NBs after GRR under LPL illumination. In panels (a,c,d), several particle images from other frames were pasted to show more NPs.

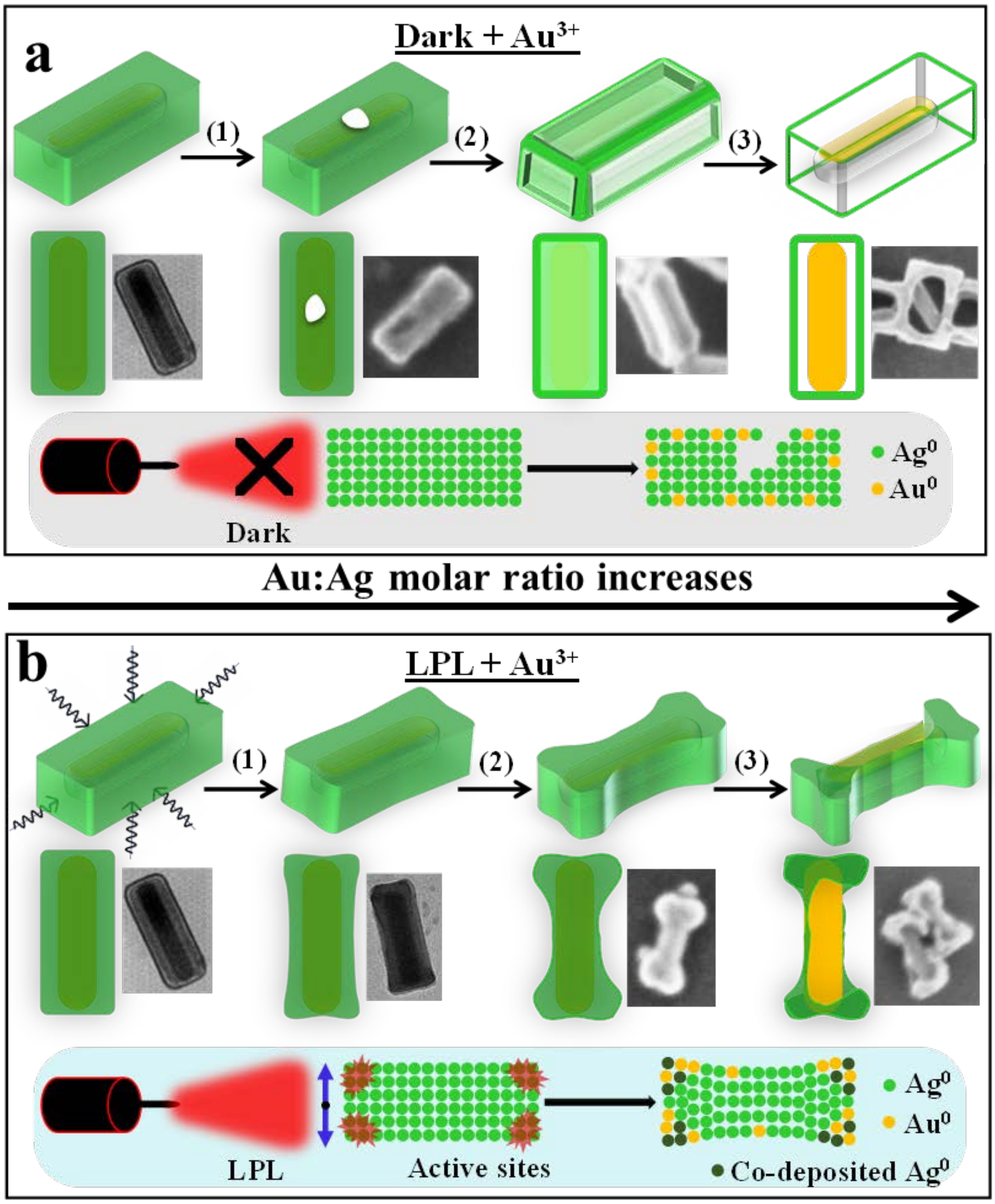

Fig. S17: Schemes illustrating the evolution of different morphologies of Au@Ag NBs (a) after GRR in the dark and (b) after GRR under LPL-illumination, with increasing the concentration of added gold ions (from step 1 to step 3). The insets at the bottom of the panels depict the schematics of the atomic configurations in the two cases (dark and LPL).

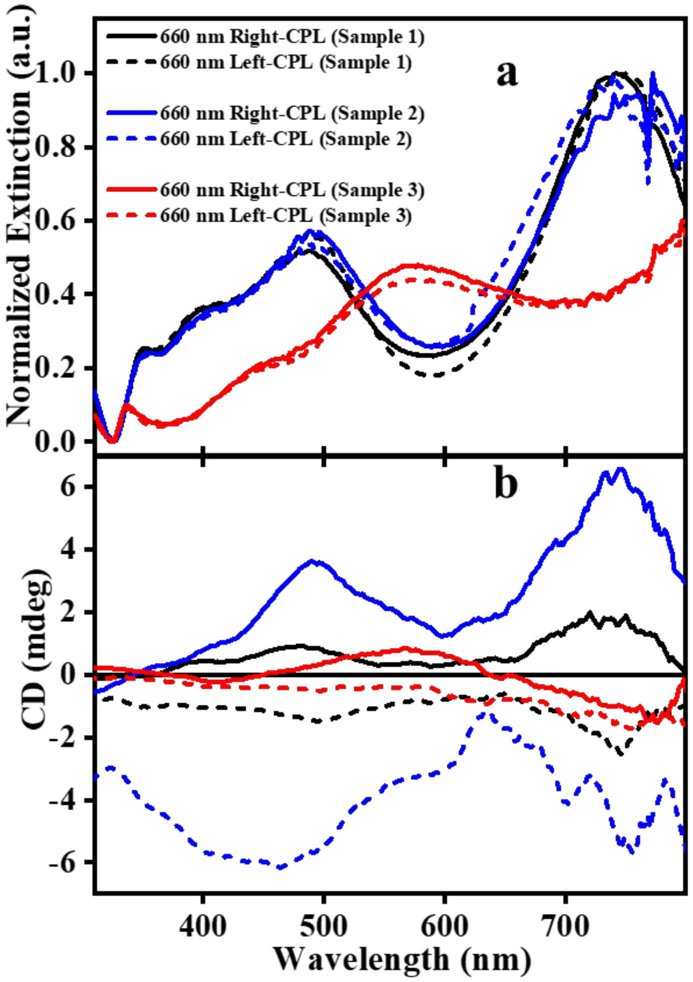

Fig. S18: (a) Normalized extinction spectra of the Au@Ag NBs (Samples 1 to 3) after GRR under right- and left-CPL illumination at 660 nm (at Au:Ag = 1:20 atomic ratio), (b) The corresponding CD spectra of the same samples.

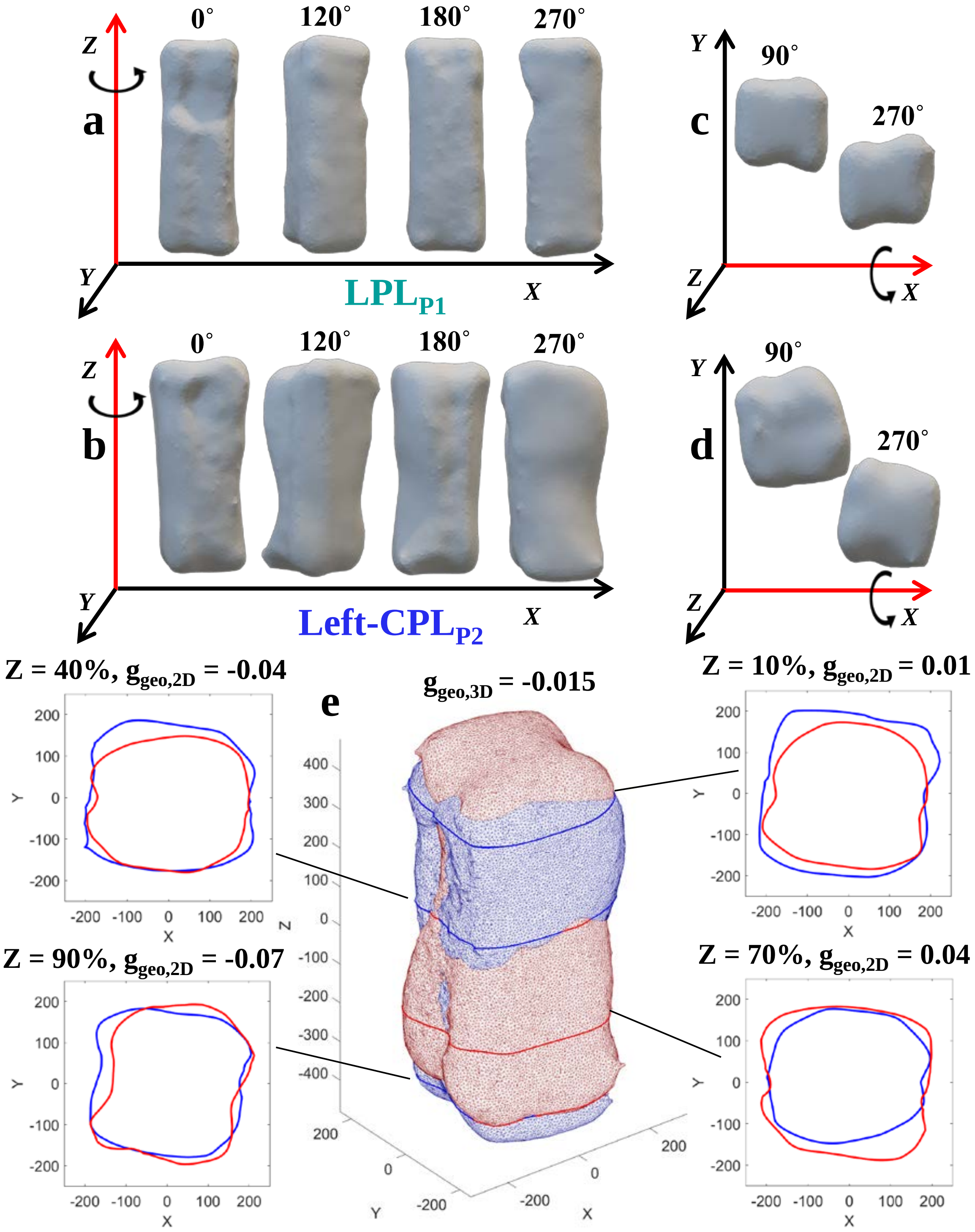

Fig. S19: Electron tomography results on post-LPL/CPL-illuminated-GRR NB morphology: Snapshots of rotated (around the long axis) projections of a 3D reconstruction of: (a) LPL illuminated GRR (LPL$_{P1}$) and (b) Left-CPL illuminated GRR (Left-CPL$_{P2}$). The experiments were done with 660 nm illumination and Au:Ag atomic ratio of 1:100. (c,d) Projections of the two ends of the corresponding NBs. (e) Geometrical analysis of chirality of electron tomography 3D reconstructed shape of a left-CPL illuminated GRR of a NB (Left-CPL$_{P2}$). The NB's shape, shown in red, was first centered with the center of mass at the axis origin, and

aligned with two principle eigenvectors of moments of inertia along Z-Y axes. Then the body was combined with a mirror image of the shape, shown in blue, inverted across the Z-Y plane. Four sections of the shape parallel to the X-Y plane are shown, demonstrating the asymmetry at four different Z-positions, with the 2D normalized chirality parameter ($g_{geo,2D}$) value in each of them. The whole 3D object's normalized chirality parameter ($g_{geo,3D}$) is also shown (see definitions in the Methods section).

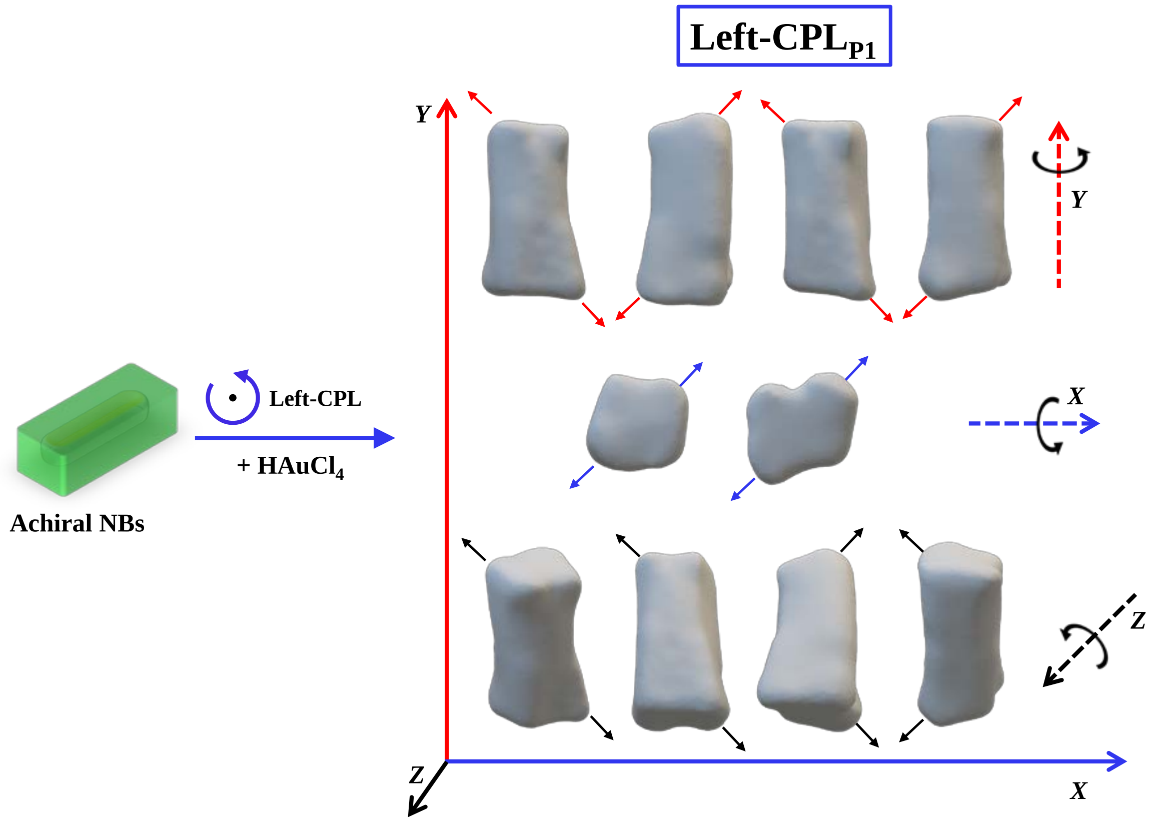

Fig. S20: Snapshots from one of the electron tomography reconstructed 3D chiral structures after GRR under left-CPL illumination at various 3D rotations. The arrows mark the corner “blobs” causing the shape chirality.

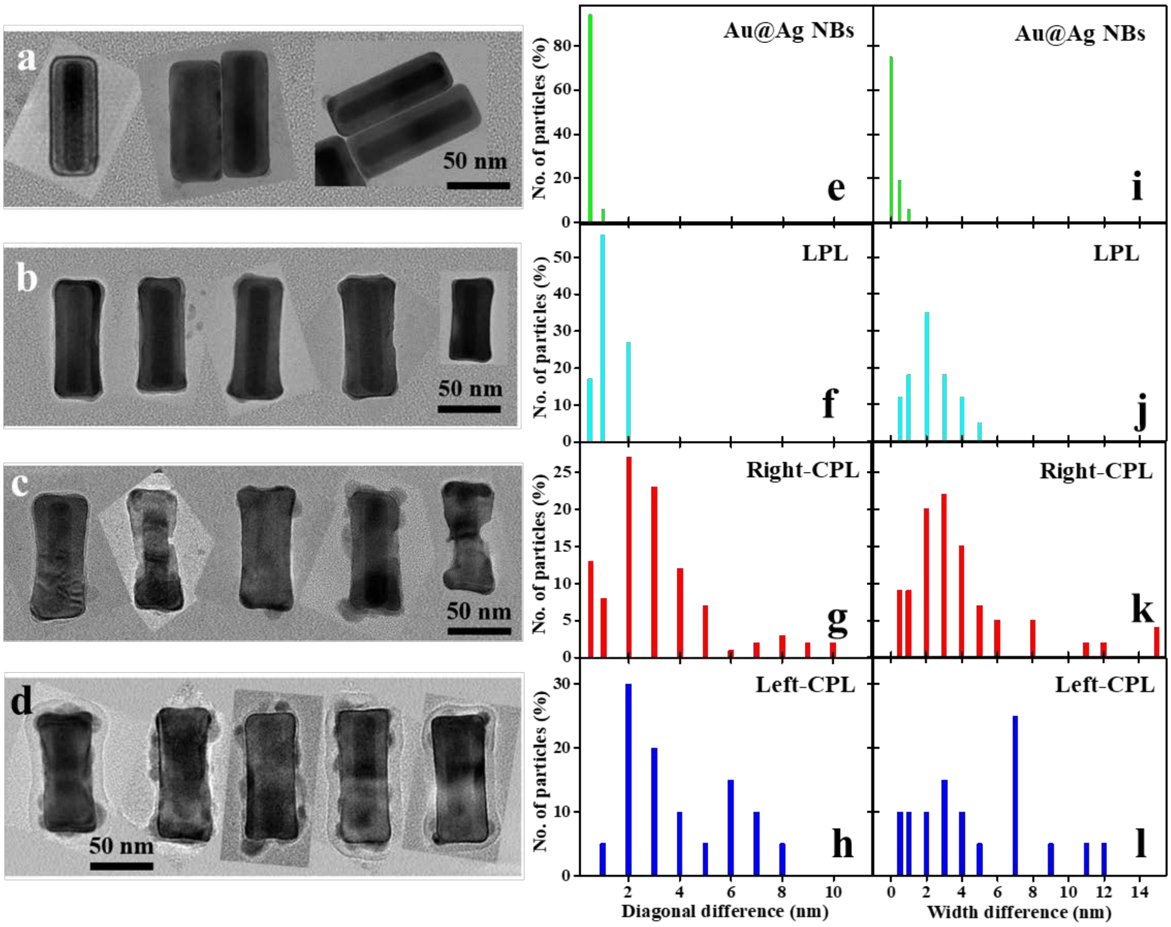

Fig. S21: TEM images of several NB samples: (a) original Au@Ag NBs, and (b) after GRR under LPL illumination at 660 nm with Au:Ag atomic ratio of 1:100, (c) right-CPL illumination, and (d) left-CPL illumination, at the same conditions as the LPL experiment. In all these panels, several particle images from other frames were collected to show 5 NPs of each sample. Histograms of the magnitude of difference between (e,f,g,h) the two diagonals and (i,j,k,l) the two end-widths in each sample, showing that the asymmetry in the cuboid shape is larger for CPL illuminated samples. About 50 particles were sampled for each histogram.

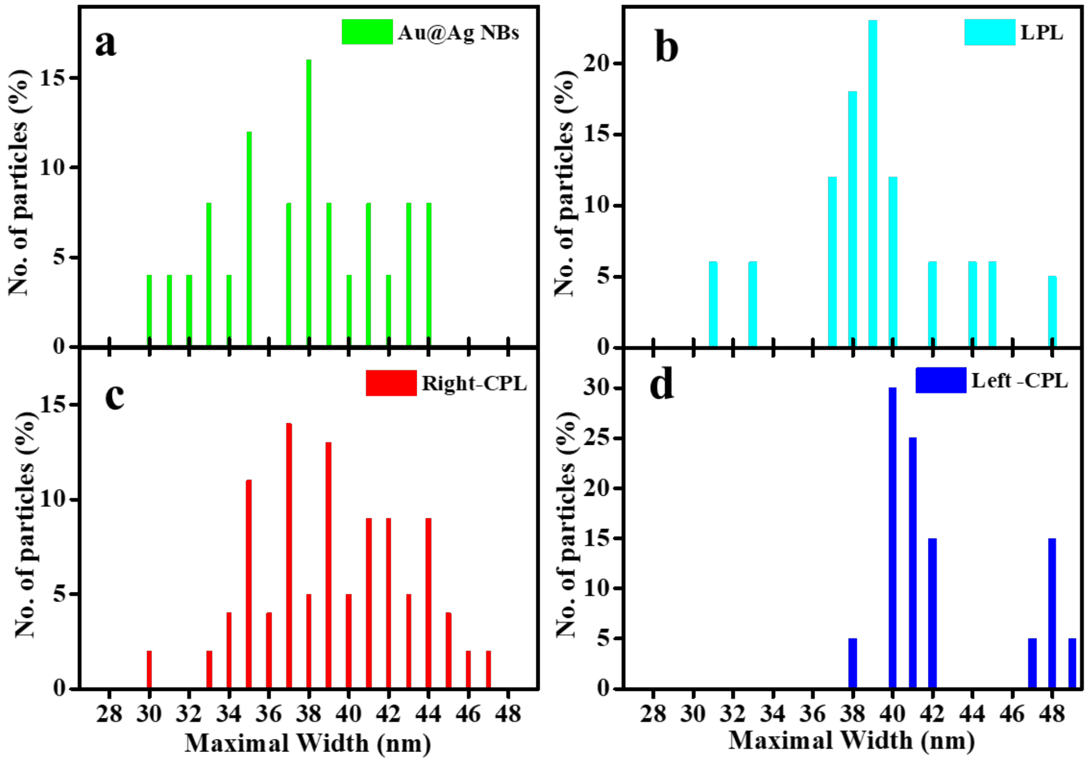

Fig. S22: (a,b,c,d) Histograms of the magnitude of maximal width in the original Au@Ag NBs, after GRR under LPL illumination, and after GRR under right- and left-CPL illumination at 660 nm with Au:Ag atomic ratios of 1:100, showing the increase in maximal width in the NBs after GRR under illumination.

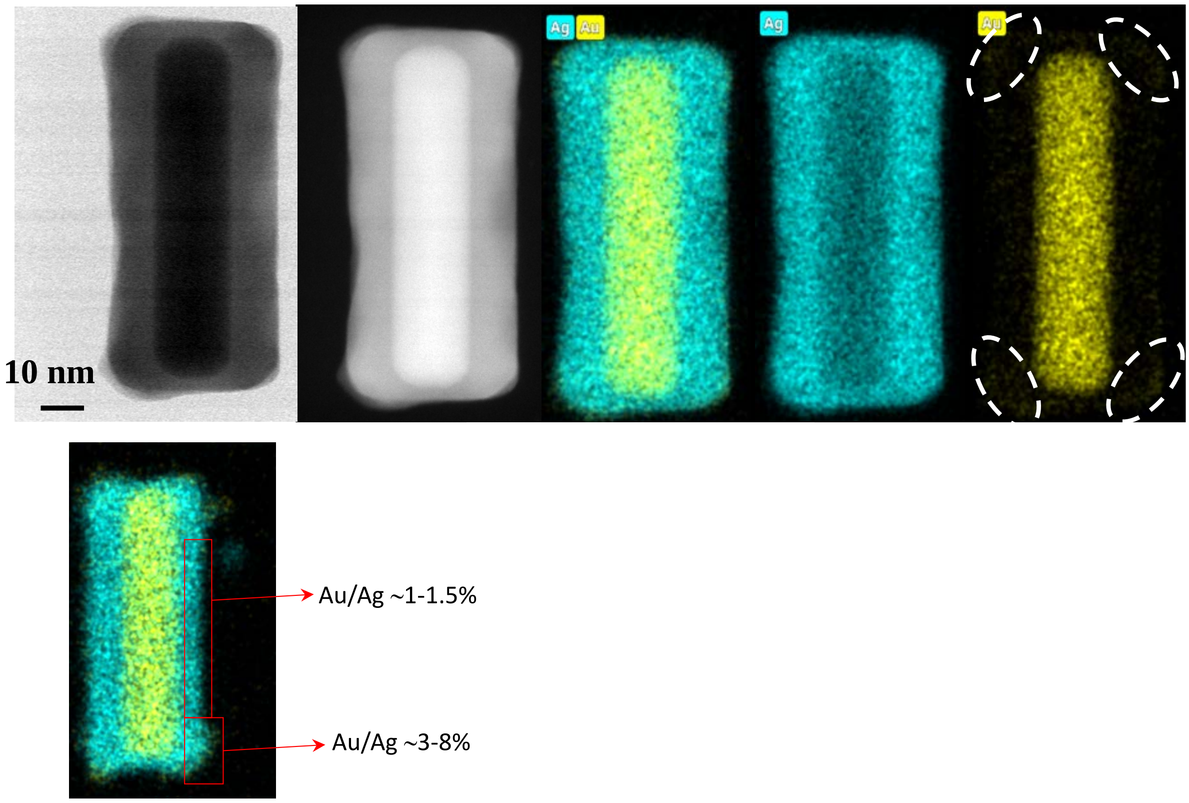

Fig. S23: (Top) STEM bright field, dark-field and EDS chemical mapping of silver and gold in one NB post-GRR under right-CPL illumination. Au:Ag ratio of GRR 1:100. The white ellipsoids mark higher concentration of gold deposited during GRR at the corners of the NB (relative to the facets). **(Bottom)** Quantification of Au:Ag ratio near the surface of the Ag shell at the corner vs. side face. The indicated numbers reflect the range of values observed over all corners and faces of the particle shown in Fig. 4 of the main text and the one shown in the top panel here.

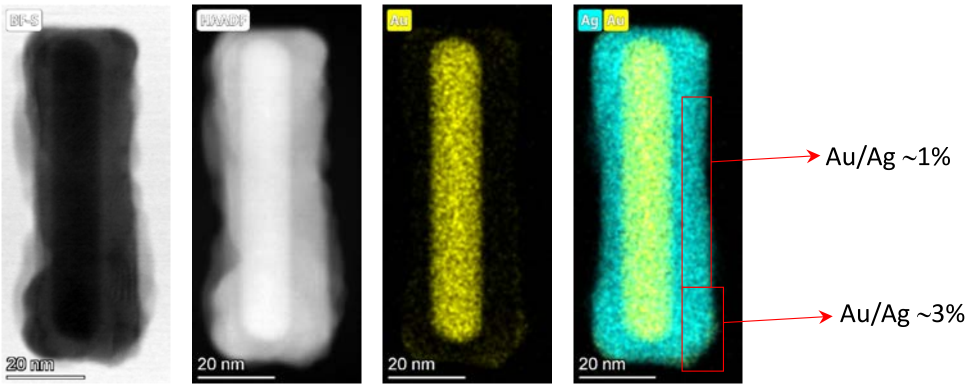

Fig. S24: STEM bright field, dark-field and EDS chemical mapping of silver and gold in one NB post-GRR, LPL illuminated at 660 nm. Au:Ag ratio of GRR 1:100. No significant difference in near surface composition relative to the CPL-illuminated case is observed.

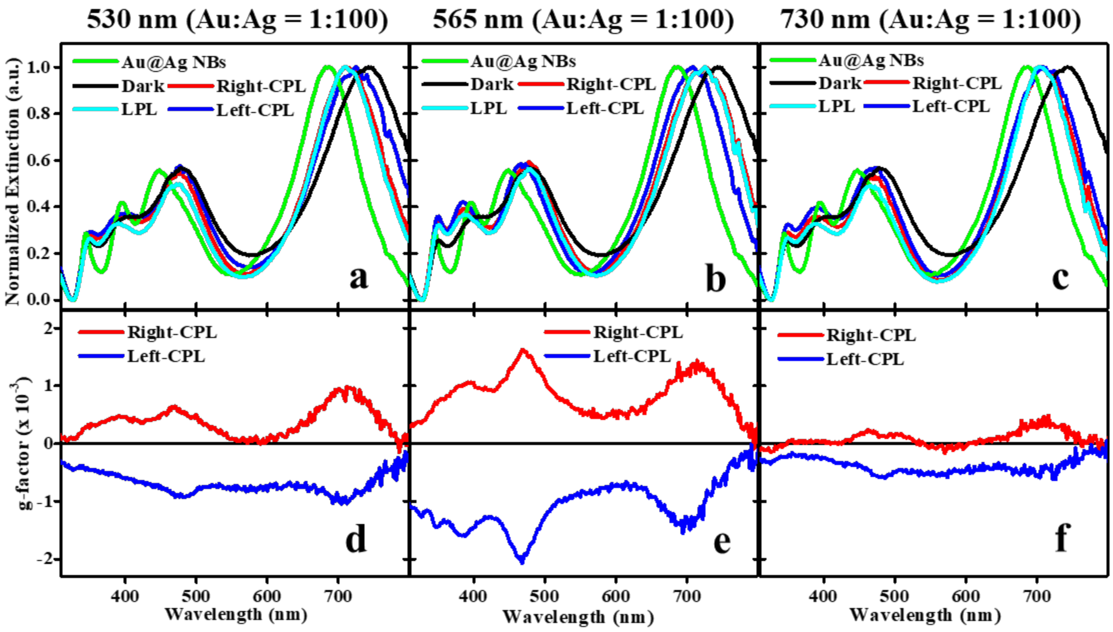

Fig. S25: (a,b,c) Normalized extinction spectra of the original Au@Ag NBs, after GRR in the dark, after GRR under LPL illumination and after GRR under left- and right-CPL illumination at different illumination wavelengths with Au:Ag atomic ratio of 1:100: (a) 530 nm, 100 mW/cm^2; (b) 565 nm, 120 mW/cm^2; (c) 730 nm, 140 mW/cm^2.

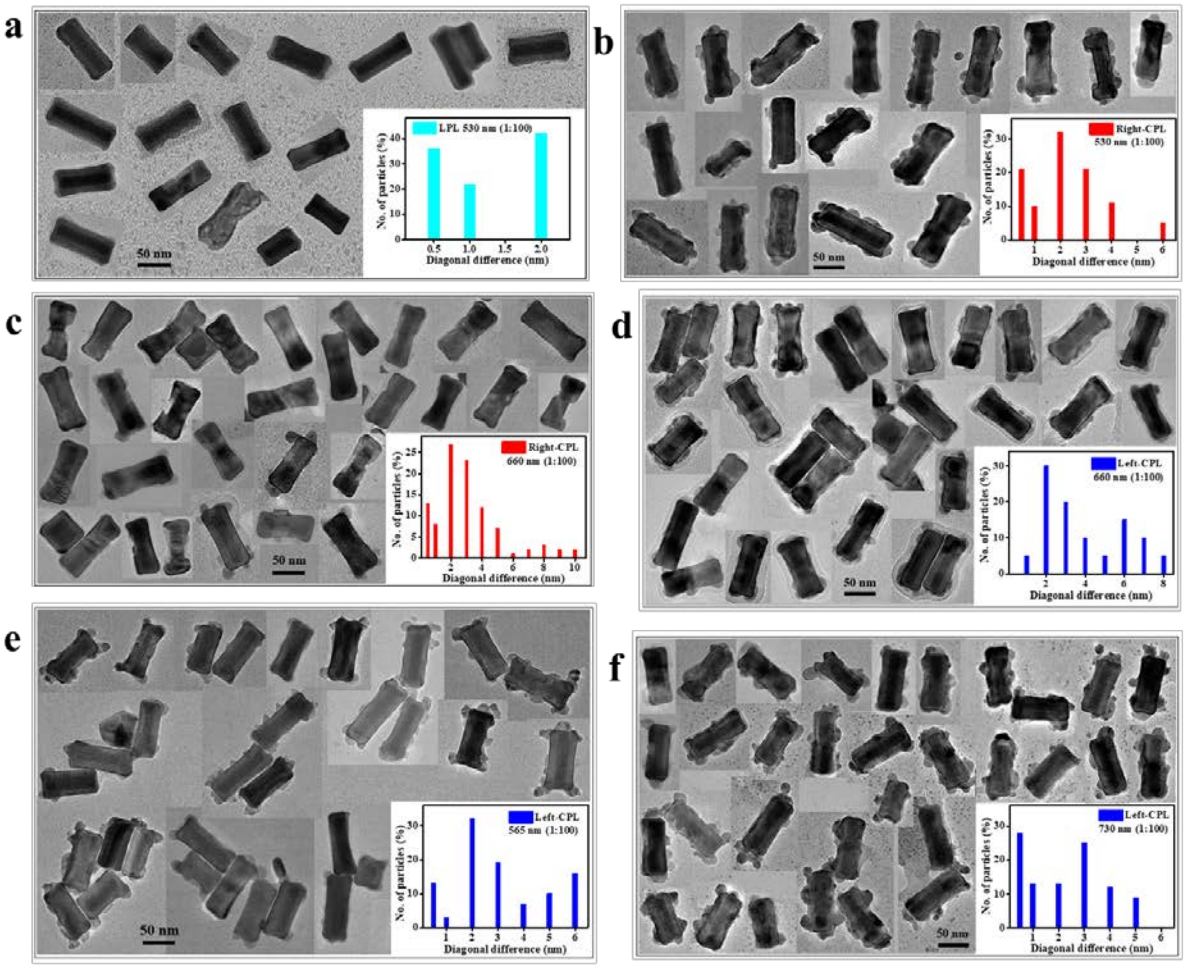

Fig. S26: TEM images of several NBs after GRR under different illumination wavelengths at Au:Ag atomic ratio of 1:100: (a) with LPL illumination at 530 nm, (b) with right-CPL illumination at 530 nm, (c) with right-CPL illumination at 660 nm, (d) with left-CPL illumination at 660 nm, (e) with left-CPL illumination at 565 nm, and (f) with left-CPL illumination at 730 nm. The insets are histograms of the magnitude of difference between the two diagonals in each sample, showing that the asymmetry in the cuboid shape is larger for CPL illuminated samples. In all TEM image panels, many NPs from different micrographs were collected together to show statistically meaningful sampling.

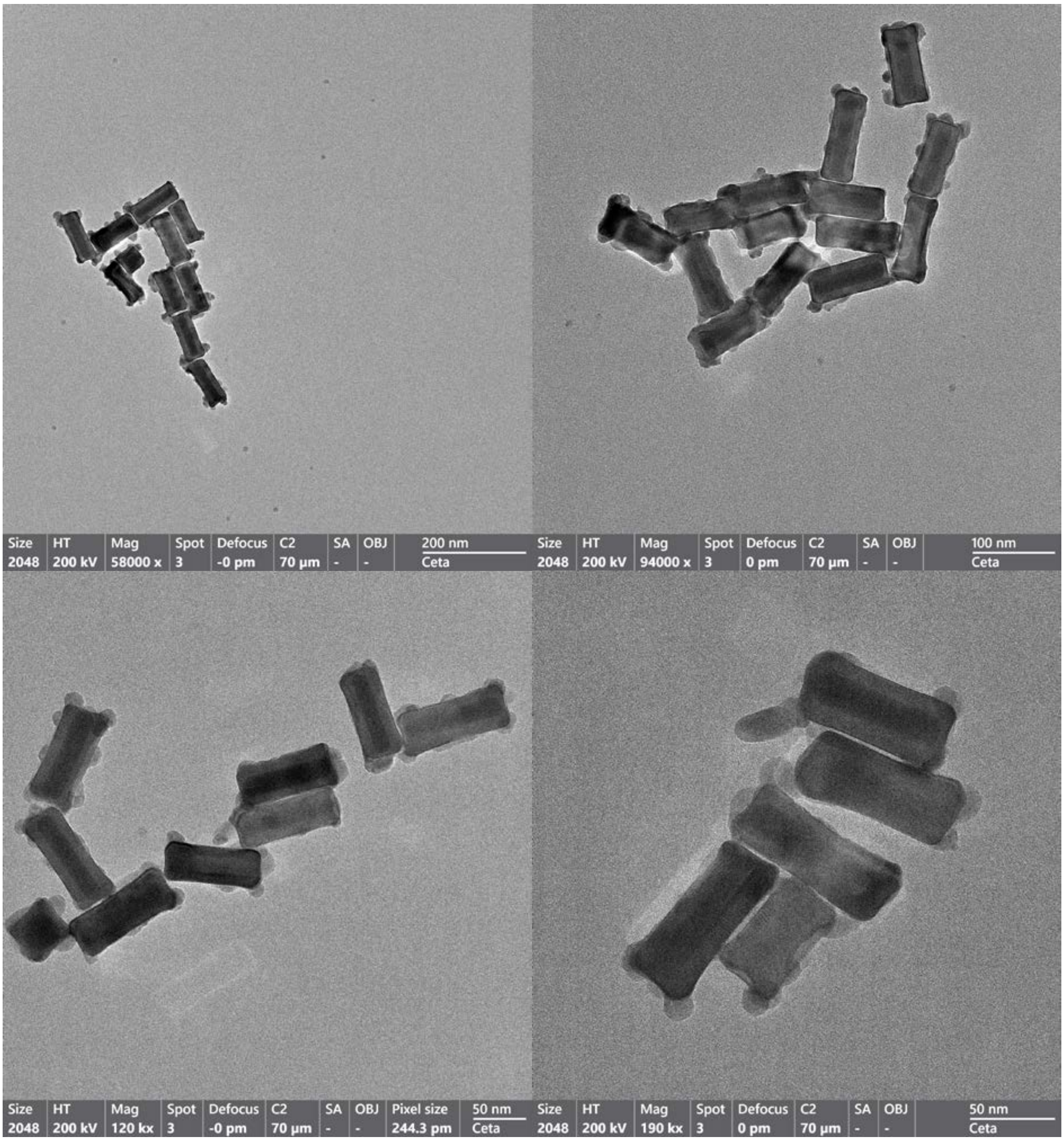

Fig. S27: TEM images of several NBs after GRR under right-CPL 660 nm illumination for Au:Ag atomic ratio of 1:100.

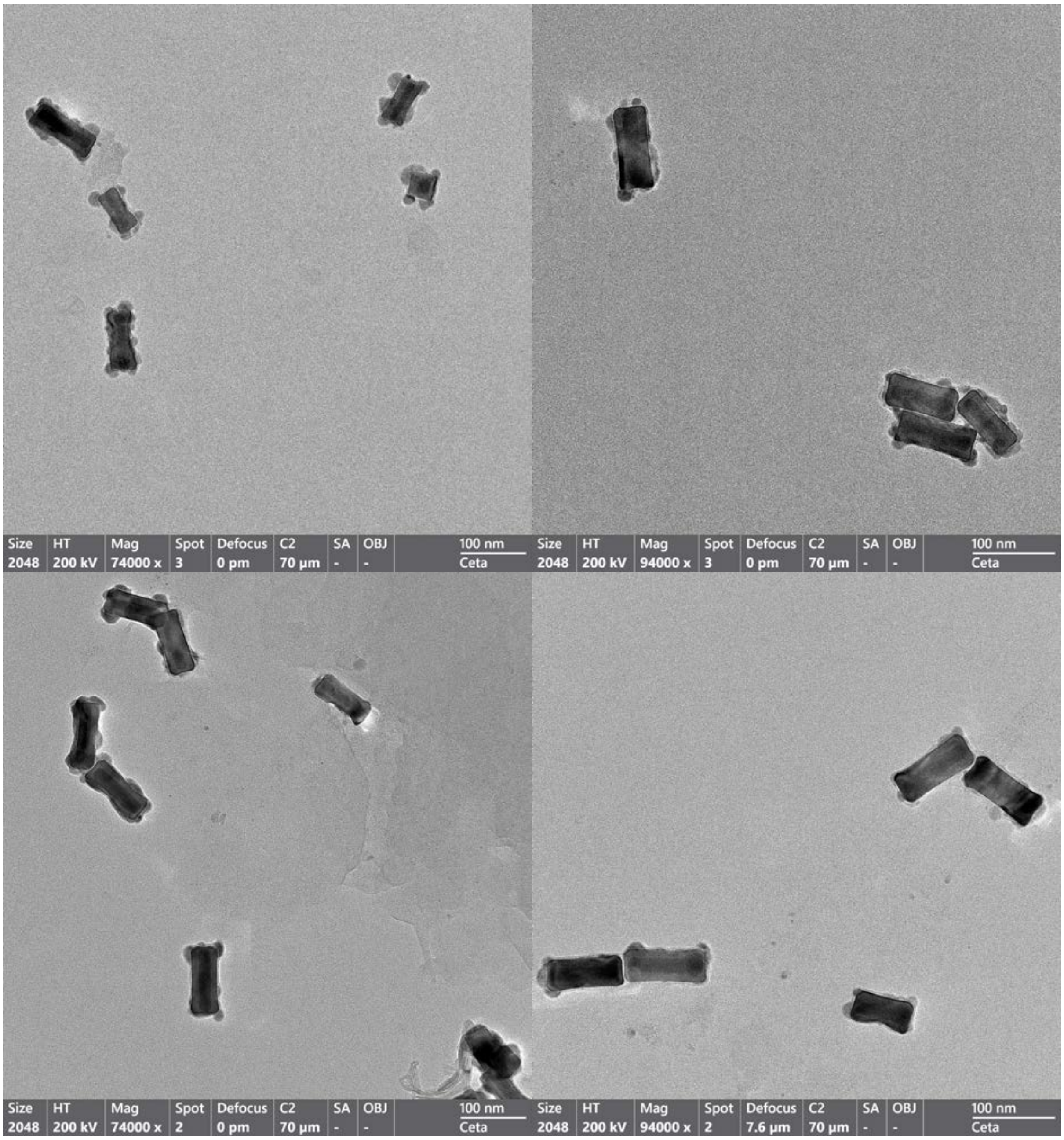

Fig. S28: TEM images of several NBs after GRR under left-CPL 660 nm illumination for Au:Ag atomic ratio of 1:100.

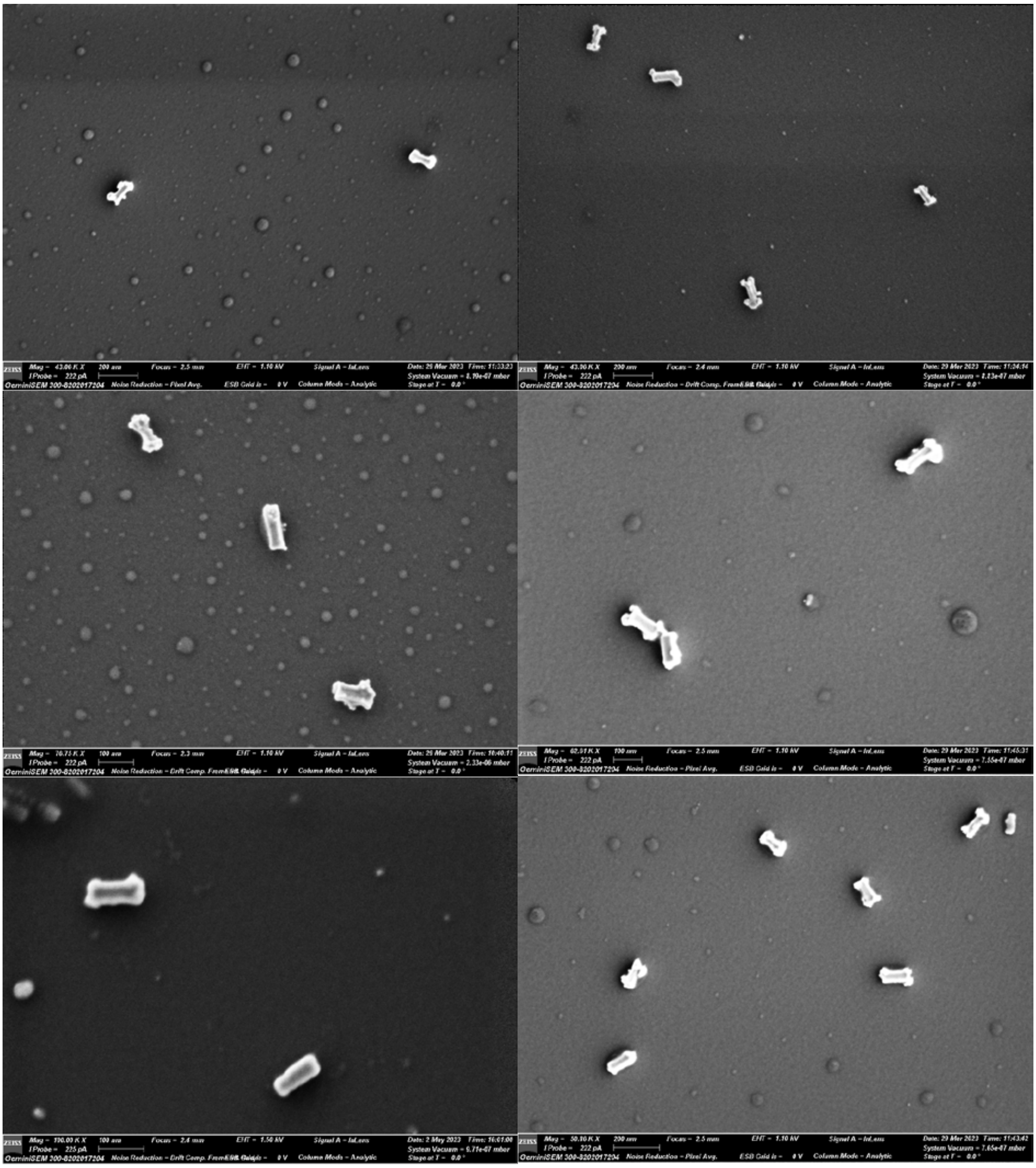

Fig. S29: SEM images of several NBs after GRR under right-CPL 660 nm illumination for Au:Ag atomic ratio of 1:100, performed in colloidal solution.

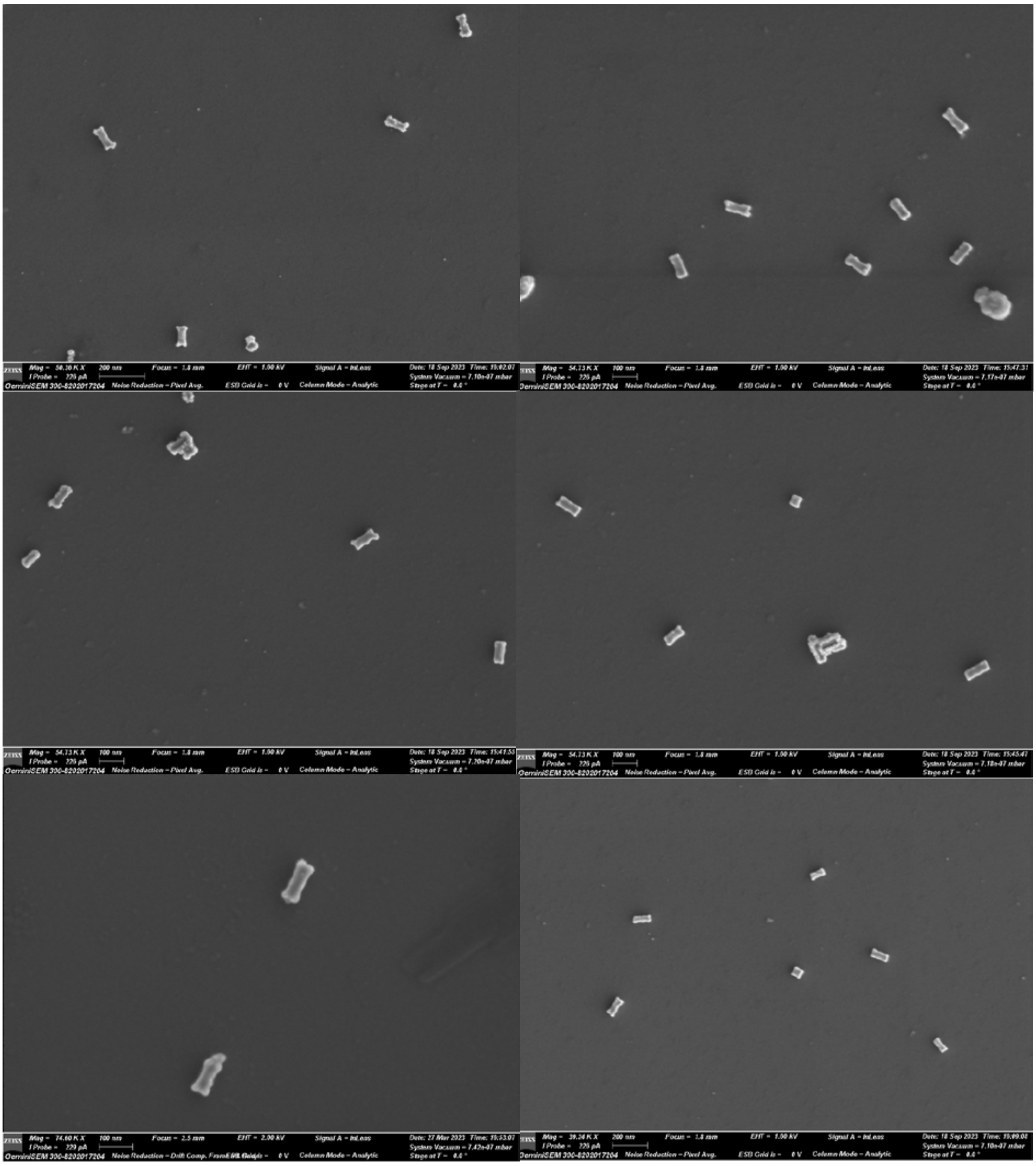

Fig. S30: SEM images of several NBs after GRR under left-CPL 660 nm illumination for Au:Ag atomic ratio of 1:100, performed in colloidal solution.

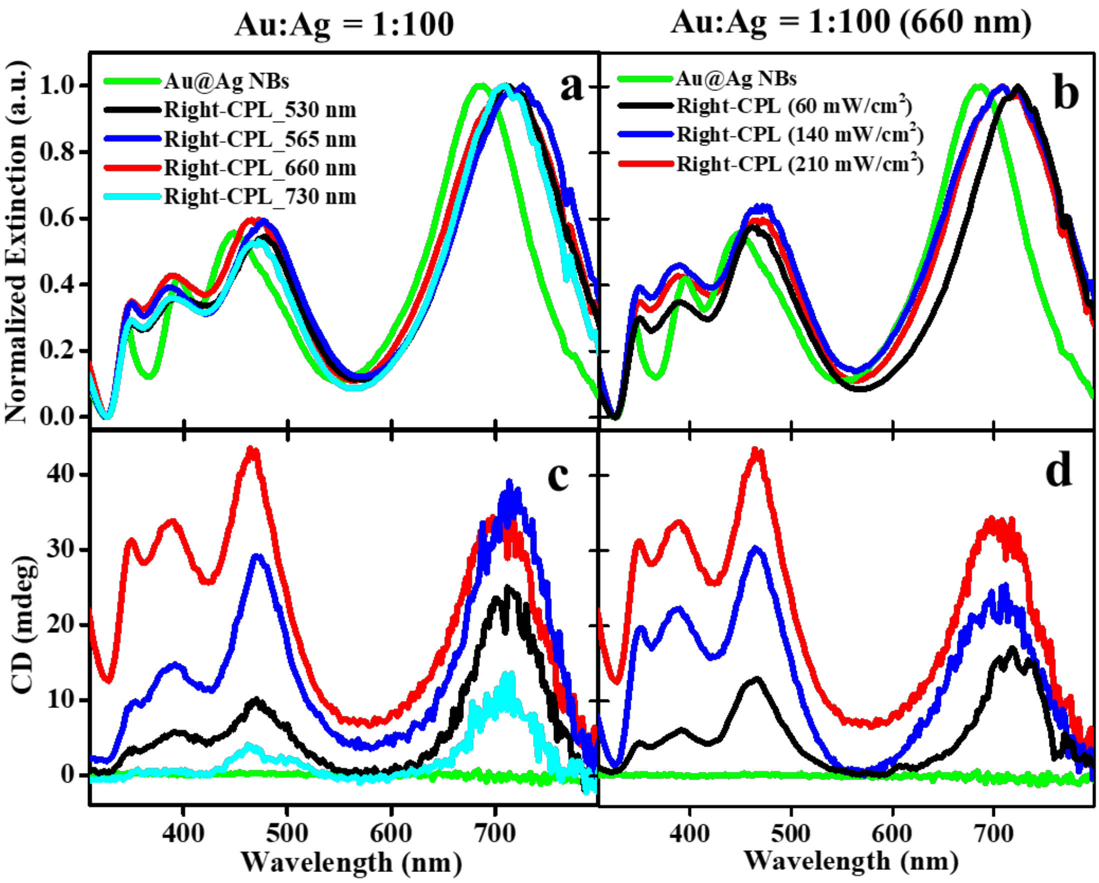

Fig. S31: (a) Normalized extinction spectra of the original Au@Ag NBs and NBs after GRR under right-CPL illumination at different illumination wavelengths (indicated in the label) with Au:Ag atomic ratio of 1:100. (b) Normalized extinction spectra of the original Au@Ag NBs and NBs after GRR under right-CPL illumination at different powers of 660 nm light, with Au:Ag atomic ratio of 1:100. (c,d) The corresponding CD spectra of the same samples.

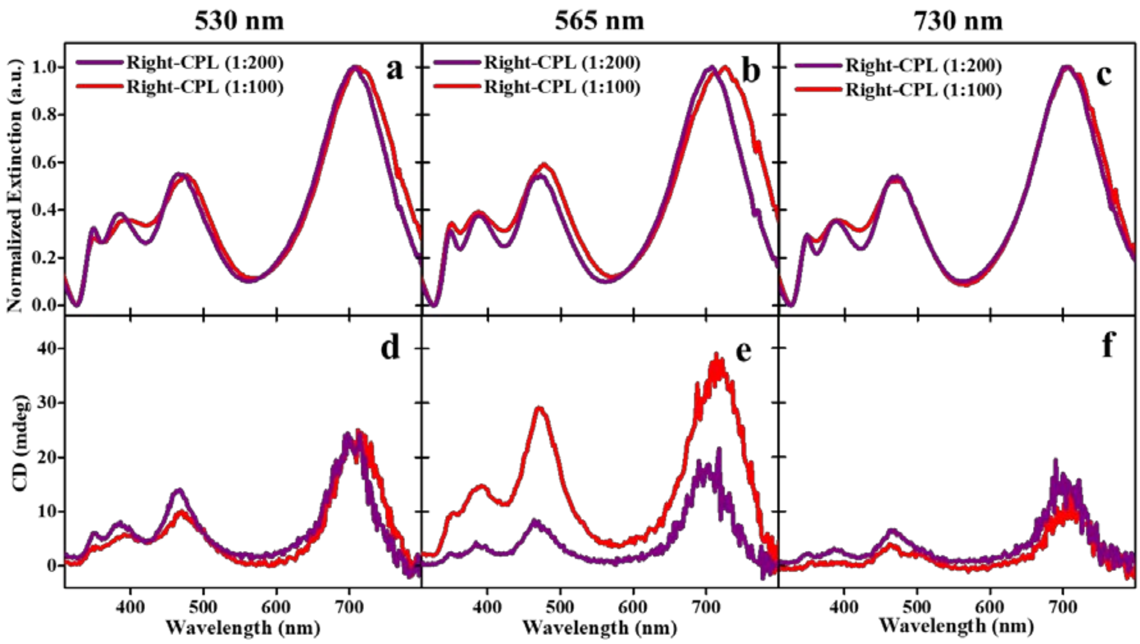

Fig. S32: (a,b,c) Normalized extinction spectra of the Au@Ag NBs after GRR under right-CPL illumination at 530 nm, 565 nm and 730 nm (with the indicated Au:Ag atomic ratios). (d,e,f) The corresponding CD spectra of the same samples.

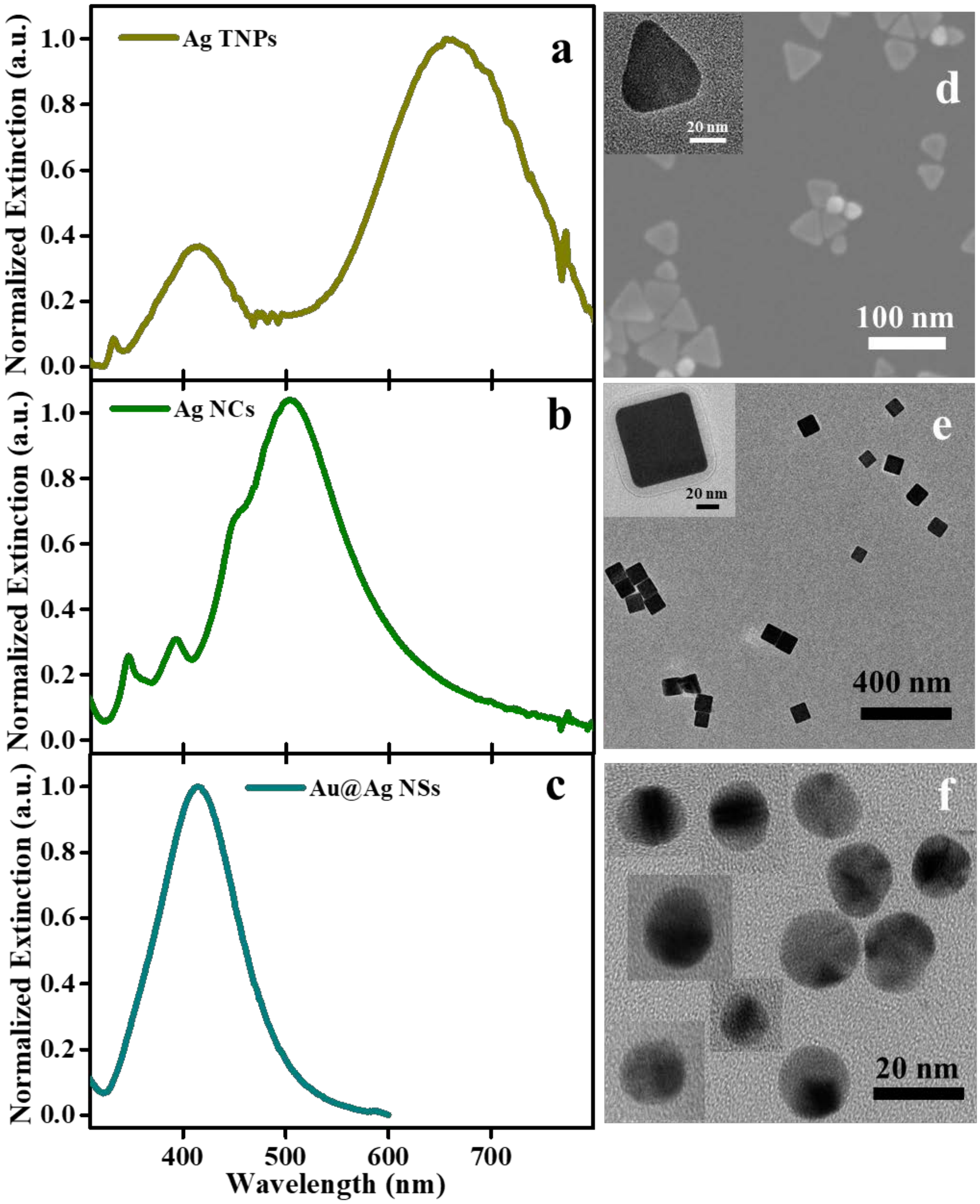

Fig. S33: (a,b,c) Normalized extinction spectra of the as prepared Ag triangular nanoprisms (TNPs), Ag nanocubes (NCs) and Au@Ag nanospheres (NSs). (d,e,f) The corresponding SEM/TEM images of the same samples.

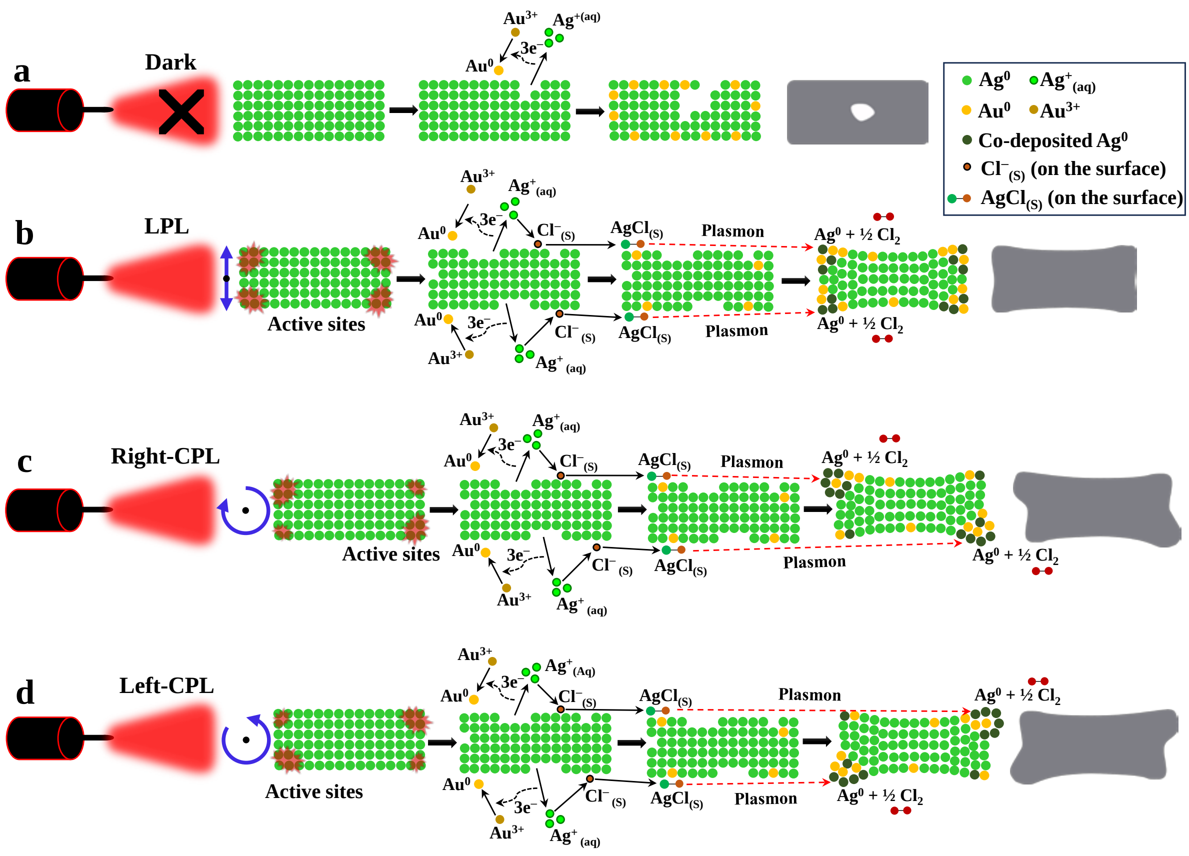

Fig. S34: Schematic illustration of the proposed plasmon-induced photochemical mechanism that generates the unique morphology of Au@Ag NBs after GRR under the different illumination conditions: (a) dark, (b) LPL illumination, and (c,d) right and left-CPL illumination.

Table S2. ICP-MS analysis of free silver and gold ion concentrations in solution after GRR under dark and right-CPL illumination at different wavelengths with Au:Ag atomic ratio of 1:100. The total concentration of Au ions taken to conduct each reaction was 5 μM.

**Wavelength**	**Au content (μM)**	**Au consumed (μM)**	**Au consumed (%)**	**Ag content (μM)**	**Molar Ratio Au:Ag**
**Dark**	2.38	2.62	52.4	7.48	1:2.85
**530 nm**	1.77	3.23	64.6	7.76	1:2.40
**565 nm**	1.79	3.2	64	7.27	1:2.27
**660 nm**	1.75	3.25	65	7.17	1:2.20
**730 nm**	1.94	3.06	61.2	7.80	1:2.54

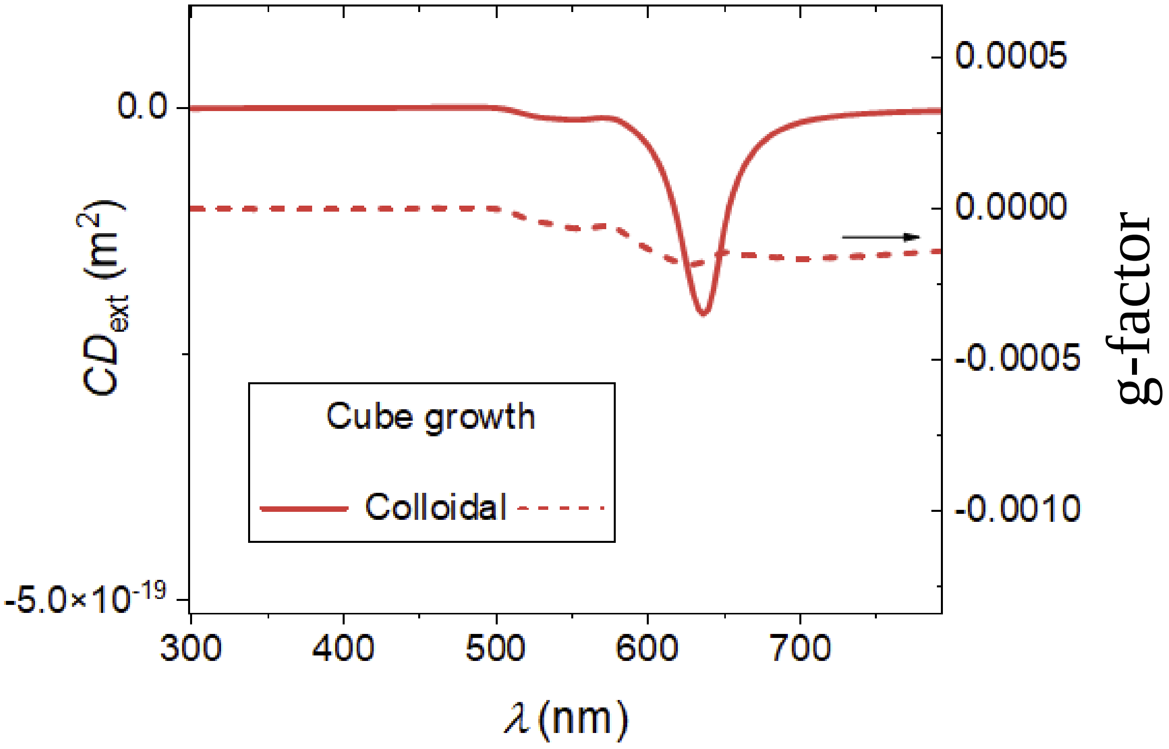

Fig. S35: Simulated CD signal of an Au nanocube grown under CPL in colloidal conditions. The solid lines represent the extinction circular dichroism, while the dashed line is their dissymmetry factor spectrum. As in the experimental case, the simulated spectra are monosignate (Adapted from Figure 3b of ref. 24).

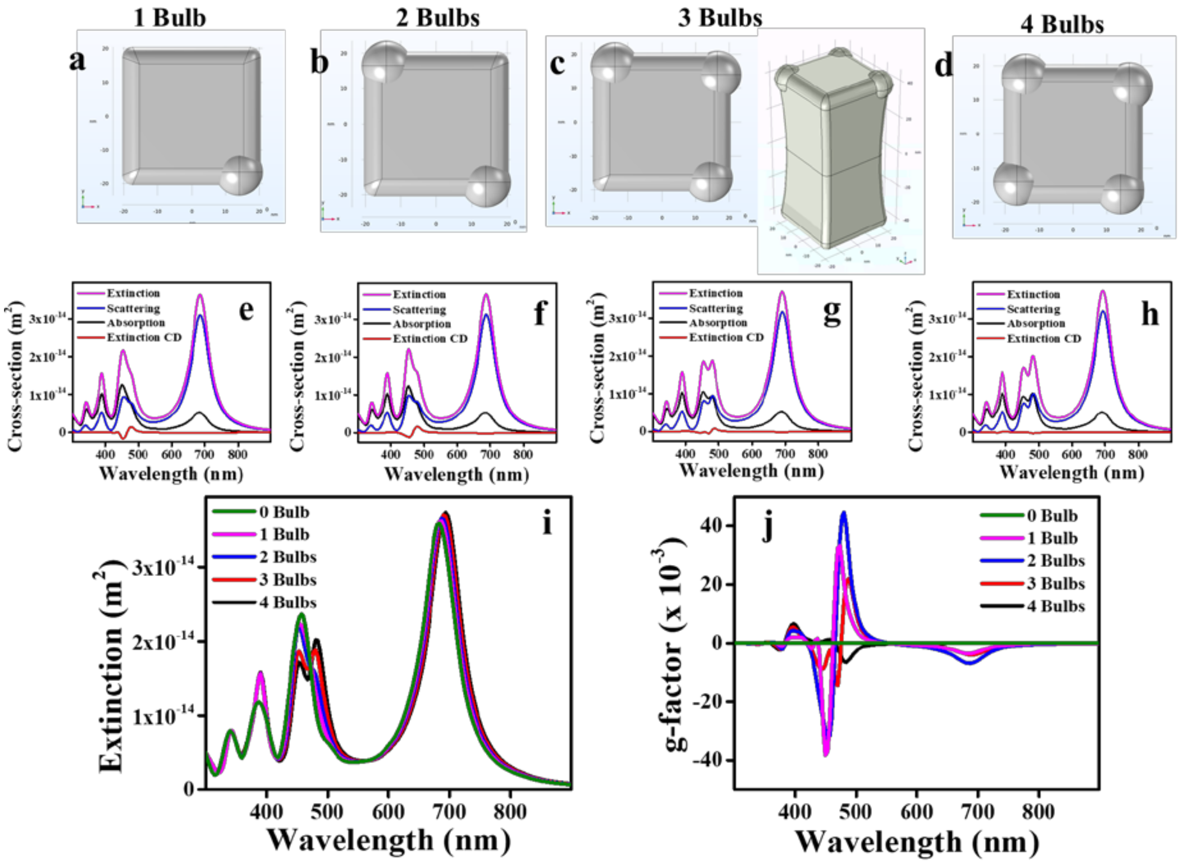

Fig. S36: The simplified model structures used to simulate CD spectra. The geometrical dissymmetry is represented by the growth of spherical “bulbs” in a chiral geometry: (a,b,c,d) Different models for the structural symmetry breaking from an achiral NB, with varying the number of bulbs affecting the chirality of the final structure. (e,f,g,h) Extinction, scattering, absorption and extinction CD spectra of the corresponding model structures. (i) Comparison of the extinction spectra of 0 to 4 bulb structures. (j) The corresponding dissymmetry factor spectra (g-factor) of the same model structures.

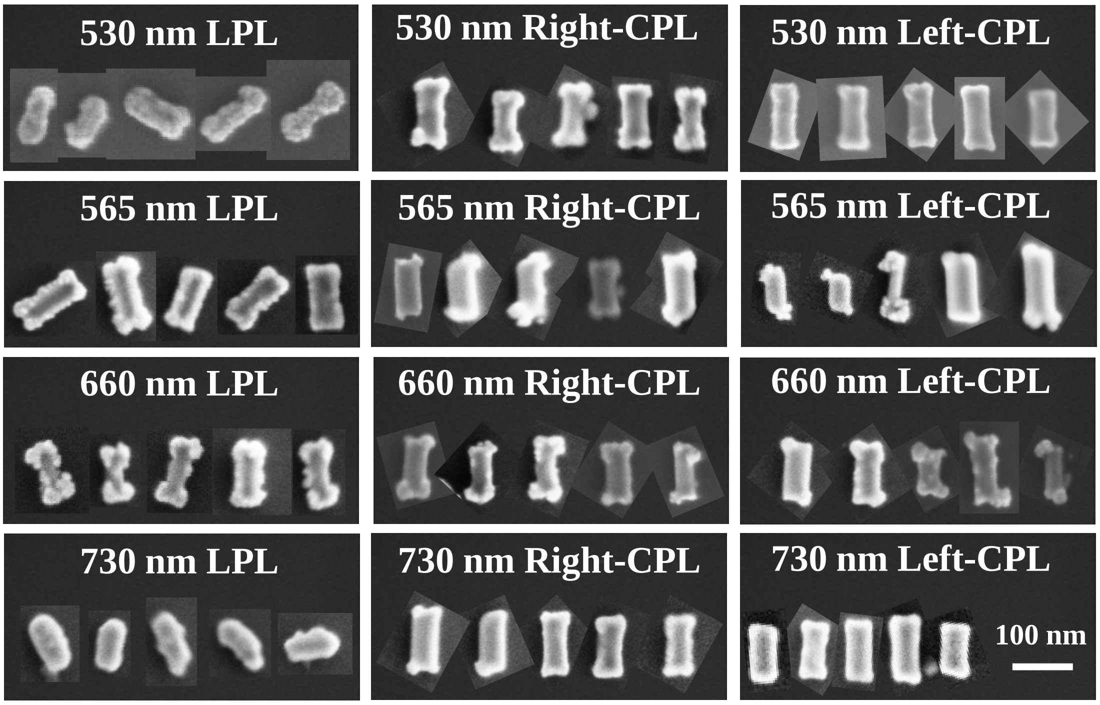

Fig. S37: SEM images of individual NBs immobilized on a silicon surface after GRR under illumination with LPL and left- and right-CPL at different wavelengths. Clear 2D chirality in opposite senses for left- and right-CPL illuminated samples can be observed for part of the NBs. In all panels, NP images were collected from several micrographs and put together to show 5 particles of each sample.
